# E2E-BPF microscope: extended depth-of-field microscopy using learning-based implementation of binary phase filter and image deconvolution

**DOI:** 10.1038/s41377-023-01300-5

**Published:** 2023-11-13

**Authors:** Baekcheon Seong, Woovin Kim, Younghun Kim, Kyung-A Hyun, Hyo-Il Jung, Jong-Seok Lee, Jeonghoon Yoo, Chulmin Joo

**Affiliations:** 1 Department of Mechanical Engineering, Yonsei University, Seoul, 03722 Republic of Korea; 2 The DABOM Inc, Seoul, 03722 Republic of Korea; 3 School of Integrated Technology, Yonsei University, Incheon, 21983 Republic of Korea

**Keywords:** Wide-field fluorescence microscopy, Imaging and sensing

## Abstract

Several image-based biomedical diagnoses require high-resolution imaging capabilities at large spatial scales. However, conventional microscopes exhibit an inherent trade-off between depth-of-field (DoF) and spatial resolution, and thus require objects to be refocused at each lateral location, which is time consuming. Here, we present a computational imaging platform, termed E2E-BPF microscope, which enables large-area, high-resolution imaging of large-scale objects without serial refocusing. This method involves a physics-incorporated, deep-learned design of binary phase filter (BPF) and jointly optimized deconvolution neural network, which altogether produces high-resolution, high-contrast images over extended depth ranges. We demonstrate the method through numerical simulations and experiments with fluorescently labeled beads, cells and tissue section, and present high-resolution imaging capability over a 15.5-fold larger DoF than the conventional microscope. Our method provides highly effective and scalable strategy for DoF-extended optical imaging system, and is expected to find numerous applications in rapid image-based diagnosis, optical vision, and metrology.

## Abstract (web summary)

The E2E-BPF microscope, a DoF-extension computational imaging platform, enabled by an end-to-end optimized binary phase filter and image reconstruction, facilitates high-resolution imaging over an extended DoF without refocusing.

## Graphical abstract

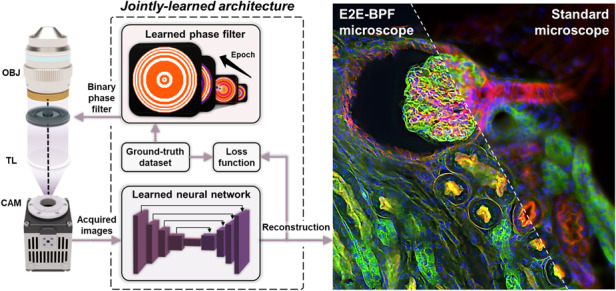

## Introduction

Microscopic imaging systems can only produce a clear image of an object within a limited depth range, known as depth-of-field (DoF). The DoF defines the depth range of an object that can be sharply imaged by a given optical imaging system, and is determined by the operating wavelength, effective focal length and aperture size of the imaging lens. In many biomedical imaging applications, such as in cytometry^1,2^, histology^3^, and endoscopy^4–6^, high-resolution imaging over a large spatial scale is often desired; for instance, a pathological examination is typically performed with a high numerical-aperture (${NA}$) objective to visualize cellular and subcellular features of tissue specimens, but it is accompanied by limited field-of-view (FoV) and DoF. Therefore, to image large-area pathological/cytology slides, either objects or imaging optics should be scanned and refocused repetitively, which is costly and labor-intensive. To enhance the DoF, various strategies have been explored over the past few decades. A simple solution would be to reduce the aperture size of the detection system as the DoF increases with $1/N{A}^{2}$; however, this inevitably causes a loss of light throughput and information capacity. Wavefront coding, combined with dedicated deconvolution methods, provides a convenient and effective route for enhancing DoF performance^7^. Various pupil filters, such as the cubic phase mask (CPM)^7^, sinusoidal^8^, logarithmic^9^, tangent^10^ phase filters and hybrid refractive-diffractive structures have been introduced for the DoF-extension and to correct for some forms of aberrations^11^. However, implementing such complex and continuous phase structures requires either expensive phase-modulating devices (e.g., spatial light modulators) or sophisticated manufacturing methods (e.g., e-beam or multi-step lithography).

Binary phase filters (BPF), composed of concentric rings with phases of 0 and π (i.e., 1, −1 in amplitude), have recently received considerable attention as DoF-extension elements owing to their simple topology and ease of manufacturing. As the object information travels through a carefully designed BPF, the resulting images can be tuned to be invariant over the desired depth range, while maintaining a high lateral resolution. In addition, owing to its discrete 0–π phase topology, BPF allows relatively simple manufacturing processes such as photolithography and thin-film deposition, making them suitable for mass production. Consequently, various BPF design methods for focus- or DoF-extension have been suggested in recent years, including exhaustive search^12–19^, analytical solutions^20–23^, and various types of optimization algorithms^24–27^. Despite these efforts, the DoF-extension performance of BPFs has not been fully explored. One of the main reasons is that, while its performance improves with an increasing number of rings^28^, developing BPF designs with more than five concentric rings is extremely challenging and computationally expensive due to the complexity of the non-linear equations involved. For multi-annulus binary filter designs involving exhaustive searching algorithms, the processing time increases exponentially with the number of concentric rings^16^. Particle swarm optimization (PSO) algorithms^29^, which are known to be effective in solving non-linear multi-dimensional problems, have been employed to design BPF by exploring the vast design space in multi-annulus binary optical elements^28,30–33^. However, PSO-based algorithms require a number of preset design parameters, and for a design task involving many parameters, the solution space is expected to grow exponentially. Moreover, PSO tends easily to fall into local optimum in high-dimensional space and has a low convergence rate in the iterative process^34^.

Here, we present a DoF-extension computational imaging platform enabled by an end-to-end optimized BPF and image reconstruction (E2E-BPF microscope). To develop BPF designs with no constraints on the number of rings, we adopted a deep learning-based end-to-end framework to jointly design the DoF-extension BPF and optimize the relevant imaging reconstruction network with a large number of datasets. The deep learning-based BPF design is enabled by introducing a penalization function in the network, which involves differentiable design variables that converge to binary states through epochs. The learned BPF was inserted into an optical microscope to produce a depth-invariant point-spread function (PSF) over the extended DoF. The resultant images were then fed into the jointly learned deconvolution network to produce high-resolution and high-contrast images over the extended DoF. We demonstrate high-resolution, high-contrast imaging capability over a >15.5× DoF of our E2E-BPF platform through numerical simulations and experiments with fluorescent beads. The biological viability of our method was further demonstrated by imaging cellular specimens and a large-scale mouse kidney tissue section stained with fluorescent dyes with no refocusing.

## Results

### E2E-BPF microscope: physics-informed, learning-based BPF design and image deconvolution

**Fig. 1:**
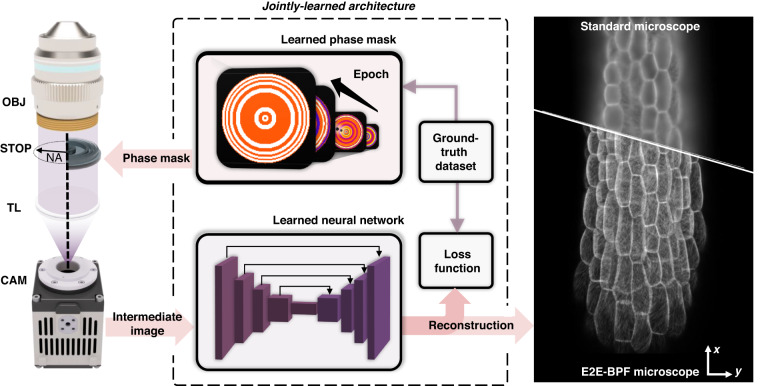
Operating principle of E2E-BPF microscopy. An axi-symmetric BPF and image reconstruction network are jointly learned through the physics-informed neural network. The numerical phantom of an Arabidopsis thaliana in three-dimension space was considered. The learned BPF is fabricated and inserted in a pupil plane in an optical microscope, which produces projected volumetric image over the extended depth range. The acquired image is subsequently fed into the jointly learned reconstruction network to generate high-resolution, high-contrast image over the extended DoF. OBJ microscope objective; NA numerical aperture; TL tube lens; CAM camera

**Fig. 2:**
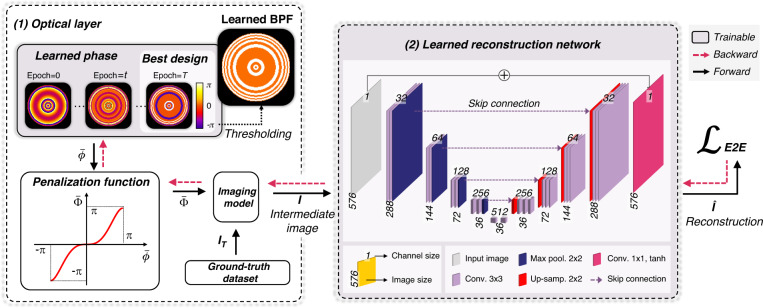
Learning pipelines for BPF and image reconstruction network. To begin, the phase filter is initialized with a continuous axi-symmetric function (e.g., axicon) and penalized by a nonlinear function that is designed to enforce the phase value in each ring to the binary states through the training process. The imaging model then predicts the image, which is then fed into the U-Net-based image reconstruction network to obtain the network output. This network output is compared against the ground-truth image, and optimization is performed to minimize the difference through a gradient-descent method

DoF for an optical microscope with a circular aperture is determined as^35^:

1 $${\rm{Do}}{{\rm{F}}}_{{\rm{clear}}}=\frac{{n}_{{medium}}\,\cdot \, \lambda }{N{A}^{2}}+\frac{{n}_{{medium}}}{M\,\cdot \, {NA}}e$$

where ${n}_{{medium}}$ is the refractive index of imaging medium, $\lambda$ is the wavelength of light, and ${NA}$ is the numerical aperture of the objective. $M$ is the magnification factor of the microscope, and $e$ denotes the pixel pitch of image sensor. The ${\rm{Do}}{{\rm{F}}}_{{\rm{clear}}}$ in our experimental setup (33×/0.75${NA}$) was estimated to be 1.19 μm. Our goal is to obtain high-resolution images over the extended DoF with jointly optimized front-end binary-phase optics and the back-end reconstruction algorithm (Fig. 1). We achieved this using end-to-end training of the BPF design and neural network as a joint optimization problem. The design process involves an evolution of both the phase filter design (i.e., the phase of each ring in the BPF parameterized by $\bar{\phi }$) and the post-processing algorithm ${\mathcal{N}}(\cdot )$ (i.e., trainable hyperparameters in ${\mathcal{N}}(\cdot )$ parameterized by ${{\mathcal{W}}}_{{\boldsymbol{net}}}$). Our proposed architecture accomplishes supervised learning using a set of ground-truth images ${I}_{T}$ to educate and evolve hardware/software variable parameters. This design pipeline and backpropagation procedure are shown in Fig. 2. The architecture consists of two major components: (1) a differentiable imaging model with a BPF to be designed, which takes in input ground-truth image and corresponding depth information $\psi$ (see Eq. 7 for the definition of $\psi$) and outputs an intermediate image ${I}$ predicted by the forward imaging model, and (2) a deconvolution neural network to produce a high-resolution, high-contrast image from the intermediate image. The optical layer simulated the image formation of a microscope with a phase filter in its pupil plane. Given a phase filter and an object with a certain defocus distance, we obtain an intermediate image ($I$) by convolving the ground-truth object information (${I}_{T}$) and the corresponding PSF. We defined the design variables as the phase values of $K$ concentric annular regions, parameterized by $K$ phase values $\bar{\phi }=({\phi }_{1},{\phi }_{2},\ldots ,{\phi }_{K})$. In our analysis, it was set to $K$ = 64 for a desired DoF of 16× that of clear aperture, as it provided a numerically accurate system response while minimizing computational cost. The same BPF design was derived for a larger $K$ (e.g., $K$ = 128) when using the same initial conditions. Detailed analysis on the number of rings for a desired DoF is provided in Sec. 1 in Supplementary. To induce the phase value to the binary states during the learning stage, a differentiable penalization function ${\mathcal{P}({\cdot})}$ was introduced within the end-to-end optimization framework (Fig. 2). The penalization function was designed to have saddle points on (-π, 0, π) to facilitate the convergence of the phase value to those values at the end of training. Finally, the BPF was obtained by taking the absolute value and threshold of the phase values. Note that the proposed penalization function accepts and produces continuous values in the range [-π, π], and the phase filter can be initialized with a generalized pupil function (e.g., Zernike functions). In our ablation study, we found that the use of phase axicon and spherical aberration as the initial conditions markedly improved optimization performance (See Sec. 2 of Supplementary). The forward imaging model performs imaging in a wide-field fluorescence microscope with a phase filter in its pupil plane to obtain the intermediate image ($I$). Then, U-Net, a widely used neural network for solving such deconvolution problems^36^, was trained to obtain the final image, which is compared against the ground-truth image (${I}_{T}$). Both the reconstruction network and phase values of the BPF are updated to minimize the end-to-end loss function ${{\mathcal{L}}}_{E2E}$ through a gradient-descent method. Our optimization problem is stated as:

2 $$\begin{array}{c}\mathop{{\rm{argmin}}}\limits_{\bar{\phi },{\boldsymbol{\mathcal{W}}}_{{\boldsymbol{net}}}}{{\mathcal{L}}}_{E2E}\\ {{\mathcal{L}}}_{E2E}={{\mathcal{L}}}_{{RMSE}}\left({\mathcal{N}}\left(I\left(\bar{\phi },\psi \right){\rm{;}}{\boldsymbol{\mathcal{W}}}_{{\boldsymbol{net}}}\right),{I}_{T}\right)+\alpha {{\mathcal{L}}}_{{BPF}}\left(\bar{\phi }\right)\end{array}$$

where the first term ${{\mathcal{L}}}_{{RMSE}}(\cdot)$ evaluates the difference between the post-processed image ${\mathcal{N}}\left(I\left(\bar{\phi },\psi \right);{{\mathcal{W}}}_{{\boldsymbol{net}}}\right)$ and the ground-truth ${I}_{T}$, and the second term ${{\mathcal{L}}}_{{BPF}}(\cdot )$ is a BPF feature loss that enforces the phase values of BPF rings to the binary states. $\alpha$ is a penalty parameter that controls the relative weight of the two terms, and it is updated through the epochs. Details of the algorithm and definitions of the loss functions are provided in the “Methods” section.

### Numerical experiments and validation

First, we validated the DoF-extension performance of the E2E-BPF microscope through numerical experiments under the same conditions as those of our experimental setup. To this end, we define the DoF of a microscope with a phase filter ${\rm{A}}$ (${{\rm{DoF}}}_{{\rm{A}}}$), as the axial range over which the structural similarity index measures (SSIMs) of the resultant images satisfy the following condition:

3 $${\rm{SSIM}}\left({I}_{T},\hat{I}(z\in {{\rm{DoF}}}_{{\rm{A}}})\right)\ge {\rm{SSI}}{{\rm{M}}}_{{\rm{thr}}}$$

Here, ${I}_{T}$ and $\hat{I}$ are the ground-truth information and the reconstructed image for the object defocused by $z$, respectively, and ${{\rm{DoF}}}_{{\rm{A}}}$ denotes the DoF obtainable with pupil ${\rm{A}}$ and the corresponding reconstruction U-Net. ${\rm{SSI}}{{\rm{M}}}_{{\rm{thr}}}$ is the threshold SSIM value, which can be set by the user. In our implementation, we set ${\rm{SSI}}{{\rm{M}}}_{{\rm{thr}}}$ to be the SSIM value at ${{\rm{DoF}}}_{{\rm{clear}}}$ = 1.19 μm for the clear aperture, which is ${\rm{SSI}}{{\rm{M}}}_{{\rm{thr}}}=$ 0.900 for our test dataset^37^. Having defined the DoF, we examined and compared the performance of the E2E-BPF microscope against those from microscopes with a clear aperture and CPM. The effective NAs were identical for all three configurations. For evaluation, images of Lenna and fluorescent tissue sections^38^ were used as ground truths, which were imaged using a microscope with a given pupil filter. We then trained the reconstruction U-Nets for each imaging condition, except for the images with the clear aperture.

**Fig. 3:**
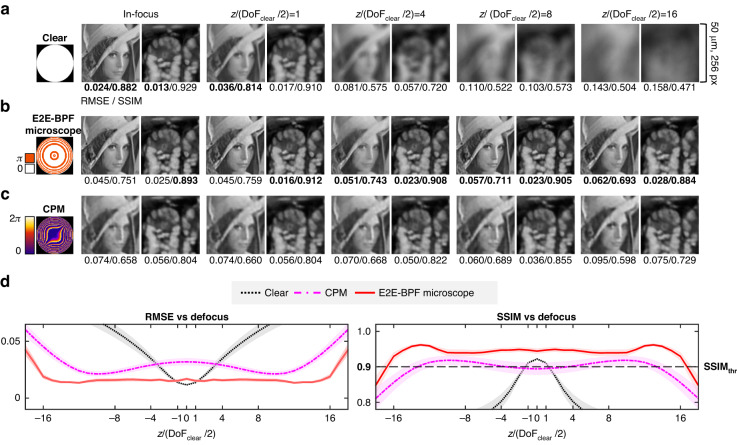
Numerical performance evaluation of E2E-BPF against clear and CPM pupil filters. The pictures of Lenna and mouse intestine tissue section were used as the reference, and numerically imaged by a microscope equipped with the filters. **a**–**c** Imaging results with clear, E2E-BPF, and CPM filters for the objects at various depth positions. Note that the results from E2E-BPF microscope and CPM were post-processed via the corresponding U-Nets optimized for each imaging condition. **d** RMSE and SSIM responses of each pupil filter as a function of defocus distance. The responses represent the mean RMSE and SSIM values evaluated over a randomly permuted test dataset. The solid lines represent the mean RMSE and SSIM values and the shaded areas represent standard error of the mean evaluated over randomly permuted test dataset (*N* = 820)

Figure 3 presents representative images at various defocus distances obtained by microscopes with clear, E2E-BPF and CPM filters in the pupil plane. Note that the defocus distances were normalized with ${\rm{Do}}{{\rm{F}}}_{{\rm{clear}}}/2$. One can easily observe that E2E-BPF and CPM provide high-contrast, high-resolution images over much larger depth ranges, whereas the image quality for the clear aperture degrades rapidly for defocus distances exceeding ${\rm{Do}}{{\rm{F}}}_{{\rm{clear}}}$(1.19 μm). We evaluated the root mean square error (RMSE) and SSIM values as a function of the normalized axial defocus distance (see Methods for the definition of image evaluation metrics) with 820 image patches from independent datasets (Fig. 3d). Consistent with the qualitative observation in Fig. 3a–c, E2E-BPF and CPM microscopes offered much higher SSIM and smaller RMSE values over a much larger DoF (19.93 μm), as compared with those of the microscope with clear aperture (1.19 μm). Compared to CPM, E2E-BPF provided higher SSIM and smaller RMSE values over the entire defocus range. In specific, as shown in Fig. 3d, SSIM values above ${\rm{SSI}}{{\rm{M}}}_{{\rm{thr}}}$(0.900) could be obtained up to $z$= ±9.96 μm for E2E-BPF (16.74× larger DoF based on Eq. 3), while those were limited to $z$= ±7.57 μm for CPM. The mean SSIM values of E2E-BPF and CPM over the entire DoF range were found to be 0.947 and 0.904, respectively. One can also note that the in-focus SSIM value of CPM (0.894) was smaller than ${\rm{SSI}}{{\rm{M}}}_{{\rm{thr}}}$, while those for clear aperture and E2E-BPF were found to be 0.922 and 0.944, respectively. We further examined the DoF-extension performance of E2E-BPF against other prior pupil designs (Table 1). We used the same datasets in Fig. 3 (i.e., images of Lenna and fluorescent tissue section as the ground-truths), and trained the reconstruction U-Nets for each imaging condition. RMSEs and SSIMs were computed for the resultant images, and the average RMSE and SSIM were evaluated for all the images in the test dataset within 20 μm DoF. The DoF-extension ratio was calculated as the ratio of ${{\rm{DoF}}}_{{\rm{A}}}$ to ${\rm{Do}}{{\rm{F}}}_{{\rm{clear}}}$. As shown in Table 1 and Fig. S3, E2E-BPF outperforms all the reference pupil designs in terms of RMSE, SSIM, and DoF-extension ability. Note that E2E-BPF jointly optimizes DoF-extension BPF and deconvolution network based on the loss function set with image metrics over a large number of images. While BPFs combined with various deconvolution algorithms^30,39^ and reconstruction networks^40^ have been proposed, E2E-BPF utilizes a significantly larger number of design variables, and thus the algorithm can explore vast spaces to obtain optimal BPF designs that produce depth-invariant PSFs over the desired DoF range. The jointly optimized deconvolution network further denoises and processes the acquired images to yield high-resolution, high-contrast images.

**Table 1:** DoF-extension performance comparisons of E2E-BPF against reference pupil designs

Pupil design	Post-processing	Scene 1 (Lenna)	Scene 2 (Mouse intestine section)	820 images from test dataset^37^	${{\rm{DoF}}}_{{\rm{A}}}$/${{\rm{DoF}}}_{{\rm{clear}}}$
		RMSE/SSIM	RMSE/SSIM	RMSE/SSIM (mean ± s.d.)	
Clear aperture	-	0.100/0.570	0.092/0.634	0.056 ± 0.034/0.766 ± 0.127	1.00
Roper et al.^27^	U-Net	0.065/0.679	0.047/0.820	0.023 ± 0.012/0.917 ± 0.059	3.64
Ryu et al.^24^	U-Net	0.084/0.648	0.078/0.742	0.036 ± 0.022/0.890 ± 0.088	4.50
Elmalem et al.^40^	U-Net	0.088/0.630	0.070/0.735	0.033 ± 0.021/0.889 ± 0.098	4.50
Milgrom et al.^18^	U-Net	0.073/0.660	0.050/0.801	0.027 ± 0.018/0.903 ± 0.088	5.58
Fontbonne et al.^30^	U-Net	0.062/0.691	0.038/0.852	0.021 ± 0.014/0.920 ± 0.077	7.83
Ren et al.^58^	U-Net	0.052/0.730	0.022/0.910	0.015 ± 0.009/0.920 ± 0.073	8.63
Ben-Eliezer et al.^15^	U-Net	0.071/0.656	0.048/0.795	0.023 ± 0.018/0.916 ± 0.080	9.14
Dowski et al.^7^	U-Net	0.068/0.672	0.046/0.831	0.028 ± 0.015/0.904 ± 0.070	13.32
**E2E-BPF**	U-Net	**0.051/0.743**	**0.022/0.912**	**0.015** ± **0.006/0.942** ± **0.020**	**16.74**

### Experimental performance evaluation: fluorescence microspheres

**Fig. 4:**
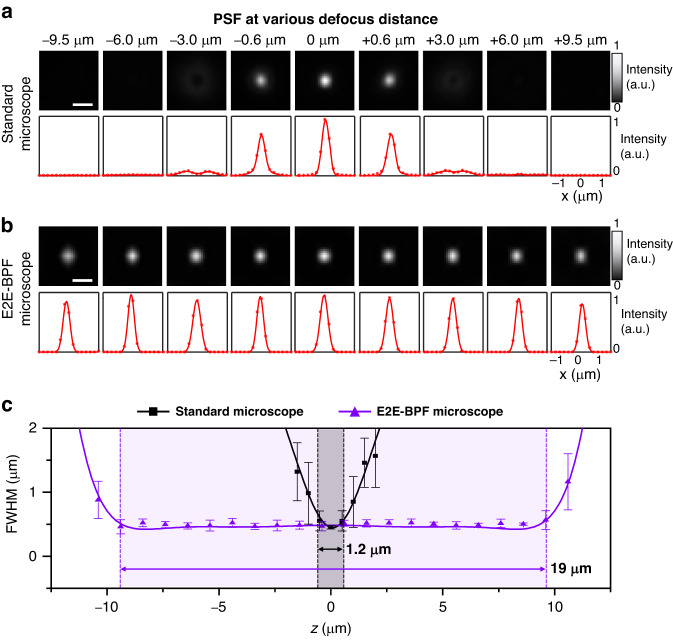
Imaging results of a fluorescent bead using standard and E2E-BPF microscopes. The top rows of **a** and **b** show the images of the fluorescent bead placed at various axial positions, and the bottom rows are the corresponding intensity profiles of the bead images, respectively. The red dots represent the raw data, and the solid curves are the results of Gaussian fitting. Scale bar in the images denotes 1 μm. **c** The graph shows the measured FWHMs of the PSFs for the standard and E2E-BPF microscopes. Each data point represents mean FWHM value, calculated from the measurements of 20 beads at 0.1 µm intervals along the depth axis. The error bars indicate standard deviation. For visual clarity, only every tenth data point is shown. It is evident that the E2E-BPF microscope provides an extended DoF while maintaining a PSF with the FWHM of 0.48 μm

We then experimentally validated the DoF-extension performance of the E2E-BPF microscope by imaging green fluorescent beads (PS-Speck Microscope point source kit 7220, Molecular Probes, USA). An E2E-BPF designed with a phase axicon as the initial condition was fabricated using photolithography, and inserted into the pupil plane of a custom-built fluorescence microscope (see Methods). The fluorescence beads were sufficiently smaller than the diffraction-limited resolution of the microscope (0.75${NA}$); thus, the image of a single bead could be considered as the PSF. We acquired images of the beads with and without the E2E-BPF in the microscope, as the monolayered beads were scanned along the optical axis in steps of 0.1 μm in the range of -12 μm to 12 μm. At each depth, we acquired 10 frames with a 100 ms exposure time, and averaged and subtracted the background to reduce noise. The images were reconstructed using the U-Net jointly optimized by the numerical simulation. Figure 4a, b shows representative images of a fluorescent bead acquired at various defocus distances with standard and E2E-BPF microscopes. For visual clarity, all the images were normalized by the peak value of the image at $z$= 0 μm for each case. In the case of the images from the standard microscope (i.e., microscope with a clear aperture), the beads became immediately blurred as they were displaced by 0.6 μm from the focal plane of the objective lens. In contrast, the E2E-BPF microscope produced high-resolution, high-contrast images of the beads over the depth range of −9.5 μm to 9.5 μm. We evaluated the full widths at half-maximum (FWHMs) of the PSFs at various depths (Fig. 4c). We performed Gaussian fitting on the intensity profiles of the bead images, and computed the FWHMs. The in-focus FWHM of the standard microscope was measured to be 0.45 μm, and it became 0.54 μm at the defocus distance of 0.6 μm. On the other hand, the PSF of the E2E-BPF microscope featured a FWHM of 0.54 μm at a defocus distance of 9.5 μm, and the mean FWHM of 0.48 μm over the range of −9.5 μm to 9.5 μm. These results indicate that the E2E-BPF microscope provides a significantly larger DoF of 19 μm, which is consistent with the estimated value of 19.93 μm in the numerical simulation.

### Experimental result of E2E-BPF microscope: monochrome fluorescent imaging

**Fig. 5:**
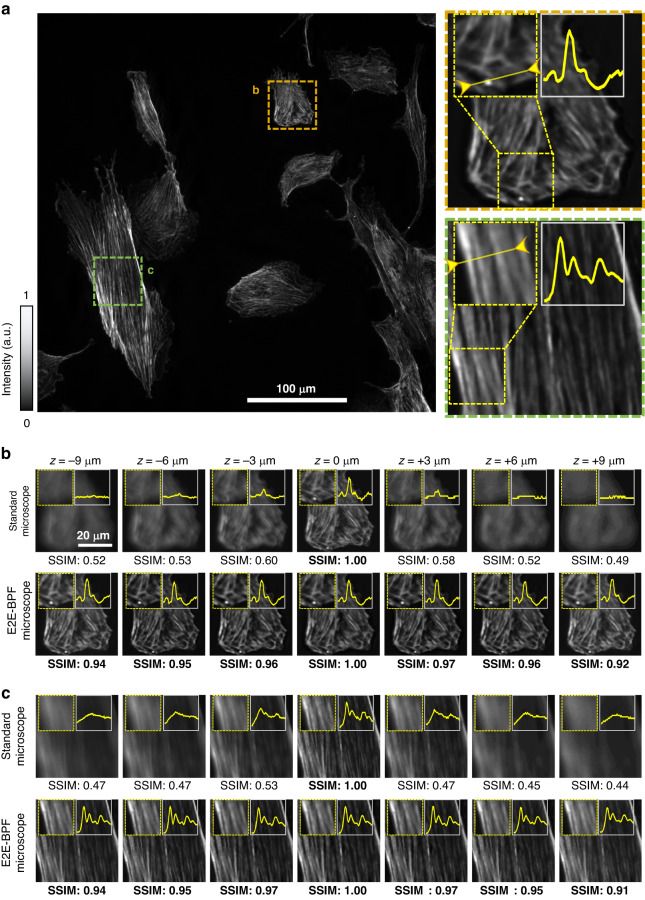
Experimental imaging results of BPAE cells labeled with red fluorescent dyes at various depth locations using standard (clear aperture) and E2E-BPF microscopes. **a** E2E-BPF microscopy image of the BPAE cells at *z*= 0 μm. **b**, **c** Magnified images of the regions marked in (**a**) at various defocus distances, along with their SSIM values

Next, we performed E2E-BPF fluorescence imaging of bovine pulmonary artery endothelial (BPAE) cells, in which mitochondria were labeled with a red fluorescent dye (MitoTracker® Red CMXRos, Life Technologies, USA). Imaging experiments were carried out using the same standard and E2E-BPF microscopes as described in the previous section. Figure 5a presents an image of the BPAE cells captured by the E2E-BPF microscope. Two regions in the imaging FoV, marked with orange and green dotted boxes were examined at various defocus distances (Fig. 5b, c). One can see that for the defocused images from the standard microscope (with clear aperture), both the image quality and the SSIM values decreased dramatically. By contrast, the E2E-BPF microscope produced the high-contrast images with high SSIM scores at all depths. Specifically, all the images from the E2E-BPF microscope featured SSIM values larger than 0.9 in the range from −9 μm to 9 μm, and the mean SSIM value was found to be 0.95. In contrast, the mean SSIM values from the standard microscopy images were measured to be 0.54.

The insets in Fig. 5b, c show the intensity profiles along the solid lines in Fig. 5a. Notably, the images from E2E-BPF microscope feature high-contrast (or high modulation depth) over the extended DoF, while the standard microscope provides high-contrast images only in the focal plane ($z$= 0 μm). Specifically, in Fig. 5b, c, the contrasts of the images from standard microscope were 0.99 and 0.98 at the focal plane, but decreased to 0.75 and 0.83 at the defocus distance of 9 µm, respectively. The mean contrast values of the images from the standard microscope over the range of −9 µm to 9 µm were found to be 0.83 and 0.88, while the mean contrast values of images from E2E-BPF microscope were 0.97 and 0.96, respectively.

### Experimental result of E2E-BPF microscope: multicolor fluorescent imaging

We further imaged a 16-μm thick mouse kidney tissue section stained with multiple fluorescent markers (FluoCells® prepared slide #3 (F24630)) to demonstrate the utility of the E2E-BPF microscope for large-area, high-throughput imaging applications. Mouse kidney tissue was stained using a combination of three fluorescent dyes: DAPI (blue) to stain the DNA, AF488 (green) to label the tubules, and AF568 (red) to visualize the F-actin filaments. Imaging experiments were performed using the same standard and E2E-BPF microscopes as in the previous section, and we employed the same image reconstruction networks for all images obtained.

**Fig. 6:**
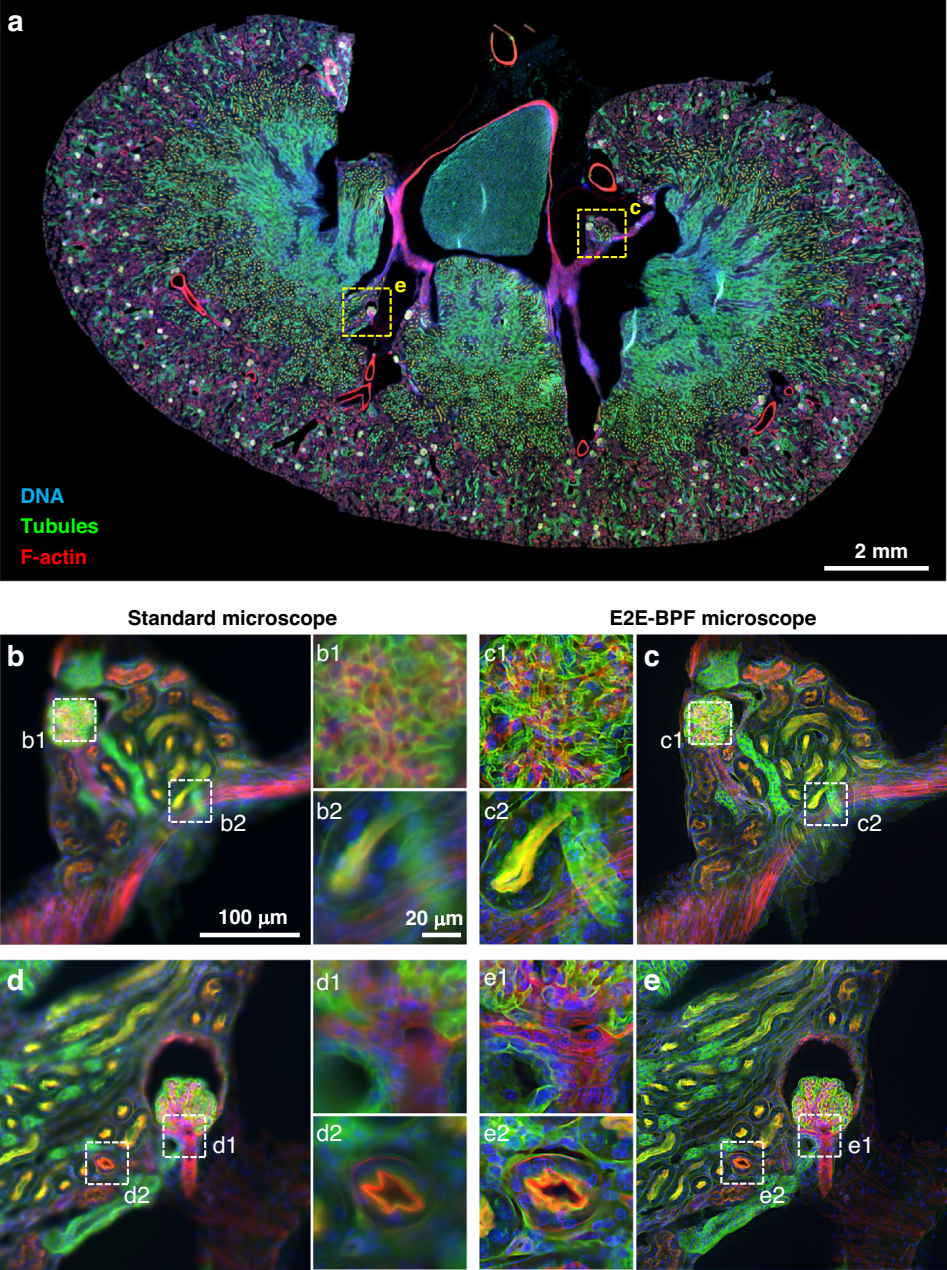
E2E-BPF microscope for multicolor fluorescent imaging. **a** Whole slide image of the mouse kidney tissue section captured with the E2E-BPF microscope. Magnified images from the standard (**b**, **d**) and E2E-BPF (**c**, **e**) microscopes of the regions are marked with yellow dashed lines in (**a**). The E2E-BPF microscope could clearly visualize the structures across various depths, without serial refocusing

Figure 6a presents a whole slide image of the mouse kidney tissue captured using the E2E-BPF microscope without serial refocusing. The image was produced by integrating 589 individual frames, each with dimensions of 2048 × 2048 pixels. The frames were stitched together with a standard image stitching algorithm^41^ with an overlap of 10% to ensure seamless integration of the frames. After the whole image was constructed, it was divided into small patches of 576 × 576 pixels, which were then inputted to the reconstruction U-Net network (See Sec. 4 of Supplementary for detailed information on U-Net). The output of the U-Net network was then reassembled into the whole slide image through a mosaic algorithm. The U-Net was capable of post-processing at a speed of 0.01 s/576 × 576 pixel patches. The total image acquisition and processing time of the E2E-BPF microscope was measured to be 30 min, which is more than 15.5 times shorter than that of a standard microscope with serial refocusing. The two regions in the image, marked with yellow dotted boxes, are shown in greater detail in Fig. 6b, d for the standard microscope and Fig. 6c, e for the E2E-BPF, respectively. Comparing the images obtained with the E2E-BPF and standard microscope, the images from standard microscope in Fig. 6b, d appear partially defocused and blurred due to its limited DoF (1.19 μm), which is much smaller than the thickness of the mouse kidney tissue section (~16 μm). In contrast, the E2E-BPF microscope provided all-in-focus images, as demonstrated in Fig. 6c, e. This difference was even more pronounced when comparing the enlarged images indicated by the white dotted box in Fig. 6b–e. Enlarged images in Fig. 6b1, c1 are glomerular regions where tubules are intricately entangled, and Fig. 6d1, e1 indicate a glomerular region with relatively low density. Due to the three-dimensional arrangement of the glomerulus at various depths, the microscope with the limited DoF produced diffuse and low-contrast images, whereas the E2E-BPF microscope could clearly image tubular and nuclei structures indicated in green and blue, respectively. Figure 6b2, c2, d2, e2 show the enlarged images of the tubule and duct regions, respectively. The nuclear and cytoplasmic fluorescence signals at various depths led to a diffuse background in the standard microscope, while the E2E-BPF microscope could clearly image structures across various depths. To validate E2E-BPF imaging on mouse kidney sections, we conducted axial scanning and quantified the local image contrast for enlarged images shown in Fig. 6b1–e1, b2–e2 (Sec. 5, Supplementary). The E2E-BPF microscope could resolve nuclei, tubules, and duct structures with a mean contrast of 0.95 for the defocus ranges considered. In contrast, the standard microscope produced partially focused images in the defocus range of 0 μm to 3 μm with a mean contrast of 0.83.

We further performed E2E-BPF imaging of 3D tumor spheroid of nominal thickness of 50 μm and compared its imaging performance against standard microscope. Details of tumor spheroid formation and imaging results are provided in Sec. 6 in Supplementary. Note that thickness of the tumor spheroid is much larger than the DoF of our E2E-BPF platform. Even for this thick specimen exceeding the DoF of the E2E-BPF microscope, E2E-BPF microscope produced the images with high contrast (>0.91), while the images from the standard microscope suffered from blurs from out-of-focus background and exhibited low image contrast (<0.71).

## Discussion

We presented a computational microscopy platform capable of high-resolution imaging of large-scale specimens over 15.5× larger DoF. We developed a data-driven, physics-informed, deep-learning architecture to jointly design and optimize a binary phase structure and image reconstruction network. We compared the imaging performance of our platform with previously reported phase filter designs and demonstrated its superior imaging performance. Experimental validations were also performed by imaging fluorescently labeled beads and tissue sections to demonstrate its validity in visualizing detailed structures across specimens without serial refocusing.

Compared to prior studies, several distinctive features should be noted in our platform: (1) Our method aims to obtain BPF designs rather than continuous phase filters. The phase filters with continuous and complex functions are often found to be challenging to fabricate. Consequently, most relevant studies have used sophisticated fabrication methods, such as e-beam lithography and multi-step photolithography, or employed active wavefront modulation devices (e.g., spatial light modulators), which are expensive and make the system bulky. In contrast, BPF is a transparent, two-state phase element; therefore, it is relatively easy to fabricate and offers amenability to mass production. Simple one-step photolithography or nanoimprinting can readily produce the designed BPF on a large scale. (2) To the best of our knowledge, our method represents the first end-to-end deep learning-based implementation of BPF and image deconvolution. The design of binary structures in a DNN framework is challenging, as it is associated with the gradient computation of binary functions. Some BPF design studies detoured this problem by approximating a binary function with some continuous functions^40^. We tackled this problem by introducing a differentiable penalization function and BPF loss term in our network, which resolves the discontinuity problem, while facilitating convergence to binary states in the final BPF design. We believe that our method is a viable design methodology for deep-learning-based binary structures in various optical applications. (3) We experimentally demonstrate the large-DoF imaging performance of E2E-BPF microscope over a broad range of the visible spectrum. Jin et al. demonstrated 5× extended DoF performance on single-color fluorescence imaging^3^ using a phase filter of continuous phase functions. Our method, on the other hand, provides much larger DoF-extension performance (15.5× compared with a clear aperture) with a binary phase filter, and demonstrated its imaging capability for both single- and multiple-color fluorescence imaging. Although the BPF was optimally designed for a single wavelength and aberration-free optical system, we demonstrated the robustness of E2E-BPF in DoF-extension to multicolor imaging. These features altogether suggest a great promise of our method in a wider range of applications in biomedical diagnosis and color vision, for example.

Our design was performed in an aberration-free microscope using a single wavelength (center wavelength of the operating spectrum), and thus any discrepancies between our model and experimental settings may contribute to the degradation of DoF. Our experimental results for the mouse kidney section stained with three fluorophores demonstrated robustness of multi-color imaging in E2E-BPF platform. However, if the fluorescent molecules exhibit emission spectra far distant from the design wavelength, the imaging performance is expected to degrade (See Sec. 7 of Supplementary). We numerically performed E2E-BPF imaging of 820 objects labeled with various fluorescent dyes (i.e., DAPI (blue), FITC (green), TRITC (red), and Cy7 (far-red)), which exhibit different emission wavelengths. The results indicate that E2E-BPF designed at 525 nm is robust to variations in emission wavelength of <110 nm, but if the spectral shift from the design wavelength exceeds 250 nm, the performance of the E2E-BPF microscope decreases.

One can consider the extension of our platform in various directions. For instance, one might incorporate the system aberration into our design framework to further enhance the image quality. The measurements of the system aberration can be performed, for example, by imaging isolated fluorescent particles across the 3D space of interest^42^. The PSFs can then be incorporated into the physical model to jointly optimize filter structure and deconvolution network. To demonstrate the viability of this aberration-informed E2E-BPF design, we performed numerical experiments (Sec. 8, Supplementary), and found that the aberration-informed E2E-BPF design outperformed aberration-ignorant BPF design in terms of both DoF and image quality. Further, this aberration-informed design strategy can be extended to handle spatially-varying aberration in 3D microscopes. In this case, an axi-symmetric BPF may not be suitable for handling spatially-varying aberrations, and one may thus need to explore more design spaces and configurations (e.g., binary or continuous phase functions). In addition, aberrations derived from possible mismatch between nominal immersion liquid and samples of imaging, which is a major source of image degradation in high-${NA}$ (i.e., ${NA}$ > 1) imaging systems, can be considered. This can potentially be addressed by incorporating more accurate scalar or vector beam propagation models (e.g., the Gibson & Lanni scalar model^43^) that better describe these imaging characteristics into the proposed framework.

In our study, we set our desired imaging depth to be 16× that of clear aperture, and performed the design using 64 design variables. It should be noted that our method is capable of generating E2E-BPF platform with further DoF-extension. To achieve this, however, the number of design variables (i.e., the number of rings in BPF in our case) should be increased, which would markedly increase the computation and training times for the BPF design and deconvolution network. We indeed performed BPF design for 24× DoF-extension with 128 design variables, and obtained the BPF design with 22.08× DoF-extension (Sec. 9, Supplementary). The design, however, required 2.5× longer computation time compared with the original 64-ring design. Moreover, the reduction of fluorescence intensity in the detector plane should be taken into account. Since BPF generates elongated PSFs in the detector region, the energy is distributed over the depth, which results in the decrease in the measured fluorescence signal. This feature has been noted by prior publications^24,44^. Depth-resolved, high-resolution imaging over extended 3D space can also be considered as a potential extension of our platform. The design framework can be tailored to produce PSFs that vary distinctively with emitter locations, and jointly optimized neural network generates high-resolution images over the entire 3D space^45,46^. Implementation of such microscopes may involve the exploration of various forms of amplitude^47,48^, phase^49,50^ or hybrid filter structures with continuous and multi-step phase functions.

In terms of applications, one of the potential applications is its utility in light sheet fluorescence microscopy, which calls for large-DoF and high light efficiency. A BPF can be designed to generate sharp and elongated excitation light sheet or focus on the illumination path^51^. The resultant fluorescence emission from a large 3D sample can be detected though our E2E-BPF platform, allowing for high light-throughput, high-resolution volumetric imaging of fluorophores without re-focusing. In our experiments, we did not observe any notable photodamage and photobleaching in longitudinal E2E-BPF imaging (Sec. 10 of Supplementary). The E2E-BPF platform is also robust in terms of axial drift because of its elongated PSF. These features are highly desirable in imaging studies that require long-term examination of dynamic features of biological specimens. Other 3D imaging modalities can also benefit from the E2E-BPF platform. For examples, optical coherence tomography^4,6,52,53^ and photoacoustic^54^ microscopy require high-resolution imaging over an extended DoF. Our BPF is expected to find its utility in enhancing the imaging performance and broadening its applications.

## Methods

### E2E-BPF design

The E2E-BPF is composed of $K$ concentric rings, with their phase values parameterized by the vector, $\bar{\phi }=({\phi }_{1},{\phi }_{2},\ldots ,{\phi }_{K})$. Each element of the vector $\bar{\phi }$ can be initialized to an arbitrary value in the range of [−π, π], but is designed to converge to the binary states, i.e., 0 or π at the end of learning. To achieve this, a differentiable penalization function ${\mathcal{P}(\cdot)}$ was applied to $\bar{\phi }$. We conceived a penalization function given as:

4 $$\bar{\Phi }={\mathcal{P}}\left(\bar{\phi }\right)=\frac{1}{7}{\bar{\phi }}^{7}-\frac{2{\pi }^{2}}{5}{\bar{\phi }}^{5}+\frac{{\pi }^{4}}{3}{\bar{\phi }}^{3}$$

which exhibits the saddling points at (−π, 0, π). Note that this penalization function is the anti-derivative of the triple-well-potential function defined in^55^. With the penalized vector $\bar{\Phi }$, the E2E-BPF phase in the pupil plane can be expressed as:

5 $${\Phi }_{{BPF}}\left(\rho \right)={\Phi }_{k}\,\,{for}\,{\rho }_{k-1}\le \rho \,< \, {\rho }_{k}\left(k=1,2,\ldots ,K\right)$$

where $\rho$ is the radial coordinate in the pupil plane that is normalized with ${NA}/\lambda$ ($0\le \rho \le 1$).

### Imaging model

Consider a planar object ${I}_{T}$, placed at a distance $z$ from the focal plane of an imaging lens. The intermediate image $I(x^{\prime} ,y^{\prime} )$ obtained by the E2E-BPF microscope can be evaluated as the convolution of the object information with the depth-dependent PSF as:

6 $$I\left(x^{\prime} ,y^{\prime} \right)={I}_{T}\left(x,y\right)\otimes h\left(x^{\prime} ,y^{\prime} ,\bar{\Phi },\psi \right)+\eta$$

where $\otimes$ denotes the convolution operation, and $h\left(x^{\prime} ,y^{\prime} ,\bar{\Phi },\psi \right)$ is the PSF that results from BPF defined by $\bar{\Phi }$ and defocus parameter $\psi$. The defocus parameter is related to the axial defocus distance $z$ as^56^:

7 $$\psi =\frac{z}{\lambda }\frac{{{NA}}^{2}}{2{n}_{{medium}}}$$

where ${n}_{{medium}}$ denotes the refractive index of the medium. $\eta$ is the noise, which is assumed to be additive Gaussian. In our simulation, Gaussian noise with a standard deviation $\sigma$ = 0.05 was applied to the normalized blurred image in the range of [0, 1].

The depth-dependent PSF in an E2E-BPF microscope can be modeled as the squared magnitude of the Fourier transform of its pupil function:

8 $$h\left(x^{\prime} ,y^{\prime} ,\bar{\Phi },\psi \right)={\left|{\mathcal{F}}\left\{P\left(\rho \right)\,\cdot \, \exp \left(-i2\pi \psi {\rho }^{2}\right)\right\}\right|}^{2}$$

where ${\mathcal{F}}$ denotes the Fourier transform operator, $\exp \left(-i2\pi \psi {\rho }^{2}\right)$ is the phase term from defocus, and the pupil function $P\left(\rho \right)$ is expressed as:

9 $$P\left(\rho \right)={\rm{circ}}\left(\rho \right)\,\cdot\, \exp \left(i{\Phi }_{{BPF}}\left(\rho \right)\right)$$

Here, ${\rm{circ}}(\rho )$ denotes a circular pupil with its radius normalized to ${NA}/\lambda$.

### Loss function

The end-to-end loss (${{\mathcal{L}}}_{E2E}$) consists of the RMSE loss ${{\mathcal{L}}}_{{RMSE}}$ and the BPF feature loss ${{\mathcal{L}}}_{{BPF}}$. First, the RMSE between two images is evaluated as:

10 $${{\mathcal{L}}}_{{RMSE}}=\frac{1}{\sqrt{NP}}{{||}{I}_{T}-\hat{I}{||}}_{2}$$

where $NP$ is the number of pixels.

To enforce the phase values of BPF to the binary states during the learning stage, BPF feature loss ${{\mathcal{L}}}_{{BPF}}$ and penalty factor $\alpha$ are introduced (see Eq. 2). The BPF feature loss function is given as:

11a $${{\mathcal{L}}}_{{BPF}}={{\Big \Vert}\frac{\partial {\mathcal{P}}\left(\bar{\phi }\right)}{\partial \bar{\phi }}{\Big \Vert}}_{2}$$

with

11b $$\frac{\partial {\mathcal{P}}\left(\bar{\phi }\right)}{\partial \bar{\phi }}={\bar{\phi }}^{2}\,\cdot\, {\left(\bar{\phi }-\pi \right)}^{2}\,\cdot\, {\left(\bar{\phi }+\pi \right)}^{2}$$

where the multiplication operator in Eq. 11-b is an element-wise multiplication. Note that starting from a small positive value for the penalty parameter $\alpha$, a gradient descent method was taken to minimize the loss function. Then, the penalty parameter was increased, and the process was repeated. Observe that, in the limit $\alpha \to \infty$, when the loss function is minimized, the penalty term converges to 0 for 10 epochs and the loss function is thereby minimized. Each epoch took ~1 h on a computer equipped with an Intel Xeon Gold 6226 R CPU and an NVIDIA RTX A6000 GPU. Over 10 epochs, the loss function progressively minimized. See Supplementary Section 4 for detailed information on the algorithm and the hyperparameters of the end-to-end network.

### Image evaluation metric

The imaging performance of the E2E-BPF microscope was evaluated by computing its SSIM. SSIM is a well-known quality metric used to measure the similarity between two images. The SSIM is defined as:

12 $${\rm{SSIM}}\left({I}_{T},\hat{I}\right)=\frac{(2{\mu }_{{I}_{T}}{\mu }_{\hat{I}}+{C}_{1})(2{\sigma }_{{I}_{T}\hat{I}}+{C}_{2})}{({\mu }_{{I}_{T}}^{2}+{\mu }_{\hat{I}}^{2}+{C}_{1})({\sigma }_{{I}_{T}}^{2}+{\sigma }_{\hat{I}}^{2}+{C}_{2})}$$

where the $\mu$ and $\sigma$ denote the mean intensity and standard deviation of an image, respectively. Note that ${\sigma }_{{I}_{T}\hat{I}}$ is the covariance between ${I}_{T}$ and $\hat{I}$. The positive values of the SSIM index are in [0,1]. A value of 0 indicates no correlation between the images, and 1 indicates that ${I}_{T}\,$= $\hat{I}$. The regularization constants ${C}_{1}$ and ${C}_{2}$ are used to avoid a null denominator, and we set ${C}_{1}\,$= ${10}^{-4}$ and ${C}_{2}\,$= $9\,\cdot\, {10}^{-4}$ as used in [57].

### Dataset

For the ground-truth datasets for training, histopathology images from the dataset^37^ taken under a 60×/0.9${NA}$ microscope were used. The high-frequency features in the ground-truth image allowed physically accurate image degradation through a simulation of the E2E-BPF microscope (with or without BPF), primarily due to PSF convolution, defocus blur, and added noise. A total of 25,000 images were randomly assigned to the training, validation, and testing sets, which contained 22,000, 2200, and 820 images, respectively. During training, the images were scaled to fit the pixel size of the E2E-BPF microscope and augmented by rotation and flipping.

### Experiment setup

The E2E-BPF microscope was built on an epi-fluorescence microscope composed of an objective lens (CFI Plan Apochromat Lambda 20×/0.75${NA}$, Nikon, Japan) and a tube lens (TTL200, Thorlabs, USA). A 4-f optical setup (ACT508-180 & ACT508-300, Thorlabs, USA) relayed the image from the microscope onto the detector plane to achieve an effective magnification of 33. The E2E-BPF was placed in the conjugate plane of the back aperture of the objective lens. For excitation, light from a high-power broadband LED (SOLIC-3C, Thorlabs, USA) passed through an excitation filter (89013, Chroma, USA), and illuminated the specimen under the Köhler illumination condition. The fluorescence signal was collected by the objective lens, transmitted through a dichroic mirror, and imaged by a camera (Zyla 4.2, 4.2 MB format, 6.5 µm pixel size, Andor, U.K.) behind an emission filter. To enable imaging of a large specimen, lateral scanning was enabled by a pair of linear motorized stages (LNR502E/M, Thorlabs, USA), which featured a maximum travel range of 50 mm × 50 mm. Each image frame covered a FoV of 0.4 mm × 0.4 mm.

### BPF fabrication

BPFs were fabricated on N-BK7 substrates using photolithography. This process enabled us to easily etch rings with lateral and depth uncertainties of a few micrometers and tens of nanometers, respectively. Under a monochromatic illumination at wavelength ${\lambda }_{0}$, the desired etching depth was determined as:

13 $$d=\frac{{\lambda }_{0}}{2({n}_{{substrate}}-{n}_{{air}})}$$

where ${n}_{{substrate}}$ is the refractive index of the material (in our case, SCHOTT N-BK7®) at ${\lambda }_{0}$. For example, at ${\lambda }_{0}\,$= 525 nm, $d$ is obtained as 509 nm. In contrast, under multicolor illumination, the etching depth was determined at the center wavelength of emission. See Supplementary Section 11 for detailed information on the experimental set-up and fabricated E2E-BPF.

### Sample preparation

Fluorescence microspheres (PS-Speck Microscope point source kit 7220, Molecular Probes, USA) with excitation/emission wavelengths of 505/515 nm (green) were used to evaluate the imaging performance. The diameter of the microspheres was estimated as 0.175 ± 0.005 µm. A small drop of the microsphere solution was placed on a microscope slide and allowed to dry. After the sample was completely dried, a small drop of mounting medium was added, and a coverslip was placed on top of the medium. The edges of the coverslip were sealed.

We used a prepared slide of BPAE cells (FluoCells® prepared slide #1 (F36924) for single-color fluorescence imaging. The mitochondria of the cells were labeled with MitoTracker™ Red CMXRos. The stained cells were fixed and mounted on a glass slide using mounting medium.

A cryostat section of mouse kidney (FluoCells® prepared slide #3 (F24630), Molecular probes, USA) with a nominal thickness of 16 µm was used for large-scale tissue imaging. The tissue specimen was stained with a combination of fluorescent dyes. Alexa Fluor® 488 wheat germ agglutinin was used to label elements of the glomeruli and convoluted tubules. Filamentous actin prevalent in glomeruli and brush border was stained with red-fluorescent Alexa Fluor® 568 phalloidin. Nuclei were counterstained with the blue-fluorescent DNA stain DAPI.

### Supplementary information

Supplementary

## Data Availability

All the data are available upon reasonable request to the corresponding author (cjoo@yonsei.ac.kr).

## References

[1] Ortyn WE (2007). Extended depth of field imaging for high speed cell analysis. Cytometry A.

[2] Meng Q, Li Y, Yu Y, Chu K, Smith ZJ (2022). A drop-in, focus-extending phase mask simplifies microscopic and microfluidic imaging systems for cost-effective point-of-care diagnostics. Anal. Chem..

[3] Jin LB (2020). Deep learning extended depth-of-field microscope for fast and slide-free histology. Proc. Natl Acad. Sci. USA..

[4] Lorenser D, Yang XJ, Sampson DD (2012). Ultrathin fiber probes with extended depth of focus for optical coherence tomography. Opt. Lett..

[5] Greene J (2023). Pupil engineering for extended depth-of-field imaging in a fluorescence miniscope. Neurophotonics.

[6] Kim J (2017). Endoscopic micro-optical coherence tomography with extended depth of focus using a binary phase spatial filter. Opt. Lett..

[7] Dowski ER, Cathey WT (1995). Extended depth of field through wave-front coding. Appl. Opt..

[8] Zhao H, Li YC (2010). Optimized sinusoidal phase mask to extend the depth of field of an incoherent imaging system. Opt. Lett..

[9] Sherif SS, Cathey WT, Dowski ER (2004). Phase plate to extend the depth of field of incoherent hybrid imaging systems. Appl. Opt..

[10] Le VN, Chen SQ, Fan ZG (2014). Optimized asymmetrical tangent phase mask to obtain defocus invariant modulation transfer function in incoherent imaging systems. Opt. Lett..

[11] Flores A, Wang MR, Yang JJ (2004). Achromatic hybrid refractive-diffractive lens with extended depth of focus. Appl. Opt..

[12] Gao XM, Gan FX, Xu WD (2007). Superresolution by three-zone pure phase plate with 0, π, 0 phase variation. Opt. Laser Technol..

[13] Sheppard CJR, Campos J, Escalera JC, Ledesma S (2008). Three-zone pupil filters. Opt. Commun..

[14] Sheppard CJR, Campos J, Escalera JC, Ledesma S (2008). Two-zone pupil filters. Opt. Commun..

[15] Ben-Eliezer E, Konforti N, Milgrom B, Marom E (2008). An optimal binary amplitude-phase mask for hybrid imaging systems that exhibit high resolution and extended depth of field. Opt. Express.

[16] Liu LB (2008). Superresolution along extended depth of focus with binary-phase filters for the Gaussian beam. J. Opt. Soc. Am. A.

[17] Diaz F, Goudail F, Loiseaux B, Huignard JP (2009). Design of a complex filter for depth of focus extension. Opt. Lett..

[18] Milgrom B, Konforti N, Golub MA, Marom E (2010). Pupil coding masks for imaging polychromatic scenes with high resolution and extended depth of field. Opt. Express.

[19] Xing JC, Kim J, Yoo H (2016). Design and fabrication of an optical probe with a phase filter for extended depth of focus. Opt. Express.

[20] Canales VF, Oti JE, Cagigal MP (2005). Three-dimensional control of the focal light intensity distribution by analytically designed phase masks. Opt. Commun..

[21] Zalevsky Z, Shemer A, Zlotnik A, Eliezer EB, Marom E (2006). All-optical axial super resolving imaging using a low-frequency binary-phase mask. Opt. Express.

[22] Sheppard CJR (2011). Binary phase filters with a maximally flat response. Opt. Lett..

[23] Cagigal MP, Oti JE, Canales VF, Valle PJ (2004). Analytical design of superresolving phase filters. Opt. Commun..

[24] Ryu S, Joo C (2017). Design of binary phase filters for depth-of-focus extension via binarization of axisymmetric aberrations. Opt. Express.

[25] Katkovnik, V., Hogasten, N. & Egiazarian, K. A novel binary and multilevel phase masks for enhanced depth-of-focus infrared imaging. In Proc. *52nd Asilomar Conference on Signals, Systems, and Computers* 386–390 (Pacific Grove: IEEE, 2018).

[26] Yang GG (1999). An optical pickup using a diffractive optical element for a high-density optical disc. Opt. Commun..

[27] Roper SWK, Ryu S, Seong B, Joo C, Kim IY (2020). A topology optimization implementation for depth-of-focus extension of binary phase filters. Struct. Multidiscip. Optim..

[28] Falcón R, Goudail F, Kulcsár C, Sauer H (2017). Performance limits of binary annular phase masks codesigned for depth-of-field extension. Opt. Eng..

[29] Kennedy, J. & Eberhart, R. Particle swarm optimization. In *Proc. ICNN’95-International Conference on Neural Networks* 1942–1948 (Perth: IEEE, 1995).

[30] Fontbonne A, Sauer H, Kulcsár C, Coutrot A, Goudail F (2019). Experimental validation of hybrid optical–digital imaging system for extended depth-of-field based on co-optimized binary phase masks. Opt. Eng..

[31] Fontbonne A, Sauer H, Goudail F (2021). Theoretical and experimental analysis of co-designed binary phase masks for enhancing the depth of field of panchromatic cameras. Opt. Eng..

[32] Fontbonne A, Sauer H, Goudail F (2022). End-to-end optimization of optical systems with extended depth of field under wide spectrum illumination. Appl. Opt..

[33] Lin J, Zhao H, Ma Y, Tan J, Jin P (2016). New hybrid genetic particle swarm optimization algorithm to design multi-zone binary filter. Opt. Express.

[34] Li M, Du WQ, Nian FZ (2014). An adaptive particle swarm optimization algorithm based on directed weighted complex network. Math. Probl. Eng..

[35] Oldenbourg, R. & Shribak, M. Microscopes. *Handbook of Optics*. I*: Geometrical And Physical Optics, Polarized Light, Components And Instruments* 3rd ed (eds Bass, M. & Mahajan, V. N.) 28.1–28.62.0 (New York: McGraw-Hill, 2010).

[36] Ronneberger, O., Fischer, P. & Brox, T. U-net: convolutional networks for biomedical image segmentation. In *Proc. 18th International Conference on Medical Image Computing and Computer-Assisted Intervention* 234–241 (Munich: Springer, 2015).

[37] Borkowski, A. A. et al. Lung and colon cancer histopathological image dataset (lc25000). Preprint at https://arxiv.org/abs/1912.12142 (2019).

[38] Nikon Microscopy, U. Triple band excitation (Dapi: FITC-TRITC). https://www.microscopyu.com/techniques/fluorescence/nikon-fluorescence-filter-sets/triple-band-excitation-filter-sets/triple-band-excitation-dapi-fitc-tritc (n. d.).

[39] Lévêque, O., Kulcsár, C. & Goudail, F. Can better performance be obtained when an imaging system is co-optimized with a nonlinear deconvolution algorithm? *Proc. SPIE 11866*, 118660A, (2021).

[40] Elmalem S, Giryes R, Marom E (2018). Learned phase coded aperture for the benefit of depth of field extension. Opt. Express.

[41] Preibisch S, Saalfeld S, Tomancak P (2009). Globally optimal stitching of tiled 3D microscopic image acquisitions. Bioinformatics.

[42] Ji N (2017). Adaptive optical fluorescence microscopy. Nat. Methods.

[43] Gibson SF, Lanni F (1992). Experimental test of an analytical model of aberration in an oil-immersion objective lens used in three-dimensional light microscopy. J. Opt. Soc. Am. A.

[44] Sheppard CJR, Hegedus ZS (1988). Axial behavior of pupil-plane filters. J. Opt. Soc. Am. A.

[45] Nehme E (2021). Learning optimal wavefront shaping for multi-channel imaging. IEEE Trans. Pattern Anal. Mach. Intell..

[46] Nehme E (2020). DeepSTORM3D: dense 3D localization microscopy and PSF design by deep learning. Nat. Methods.

[47] Levin A, Fergus R, Durand F, Freeman WT (2007). Image and depth from a conventional camera with a coded aperture. ACM Trans. Graph.

[48] Veeraraghavan A, Raskar R, Agrawal A, Mohan A, Tumblin J (2007). Dappled photography: mask enhanced cameras for heterodyned light fields and coded aperture refocusing. ACM Trans. Graph.

[49] Pavani SRP (2009). Three-dimensional, single-molecule fluorescence imaging beyond the diffraction limit by using a double-helix point spread function. Proc. Natl Acad. Sci. USA.

[50] Shechtman Y, Sahl SJ, Backer AS, Moerner WE (2014). Optimal point spread function design for 3D imaging. Phys. Rev. Lett..

[51] Ryu S (2020). Light sheet fluorescence microscopy using axi-symmetric binary phase filters. Biomed. Opt. Express.

[52] Zhao JJ (2022). Flexible method for generating needle-shaped beams and its application in optical coherence tomography. Optica.

[53] Liu LB, Liu C, Howe WC, Sheppard CJ, Chen N (2007). Binary-phase spatial filter for real-time swept-source optical coherence microscopy. Opt. Lett..

[54] Cao R (2023). Optical-resolution photoacoustic microscopy with a needle-shaped beam. Nat. Photon..

[55] Takezawa A, Nishiwaki S, Kitamura M (2010). Shape and topology optimization based on the phase field method and sensitivity analysis. J. Comp. Phys..

[56] Vettenburg T (2014). Light-sheet microscopy using an Airy beam. Nat. Methods.

[57] Wang Z, Bovik AC, Sheikh HR, Simoncelli EPP (2004). Image quality assessment: from error visibility to structural similarity. IEEE Trans. Image Process.

[58] Ren JH, Han KY (2021). 2.5-D microscopy: fast, high-throughput imaging via volumetric projection for quantitative subcellular analysis. ACS Photonics.

