# Supplementary material for: E2E-BPF microscope: extended depth-of-field microscopy using learning-based implementation of binary phase filter and image deconvolution
Source: Light Sci Appl. 2023 Nov 13;12:269. doi: 10.1038/s41377-023-01300-5 (PMC10641084; doi:10.1038/s41377-023-01300-5)
Supplement: Supplementary file 1 — Supplementary [file 41377_2023_1300_MOESM1_ESM.docx]

**Supplementary Information for
E2E-BPF microscope: Extended depth-of-field microscopy using learning-based implementation of binary phase filter and image deconvolution**

Baekcheon Seong^1^, Woovin Kim^1^, Younghun Kim^1^, Kyung-A Hyun^1^, Hyo-Il Jung^1,2^, Jong-Seok Lee^3^, Jeonghoon Yoo^1^, and Chulmin Joo^1,*^

^1^ Department of Mechanical Engineering, Yonsei University, Seoul, 03722, Republic of Korea

^2^ The DABOM Inc. Seoul, 03722, Republic of Korea

^3^ School of Integrated Technology, Yonsei University, Incheon 21983, Republic of Korea

^*^ Corresponding author’s Email: [cjoo@yonsei.ac.kr](mailto:cjoo@yonsei.ac.kr)

#### Table-of-contents

[Section 1 | Desired DoF vs. number of design variables 3](#_Toc138982471)

[Section 2 | Evaluated imaging performance of various E2E-BPF structures designed with different initial conditions 5](#_Toc138982472)

[Section 3 | Numerical evaluations on the imaging performance of E2E-BPF against reference pupil designs 6](#_Toc138982473)

[Section 4 | Learning-based implementation of E2E-BPF filter and reconstruction U-Net 8](#_Toc138982474)

[Section 5 | Experimental robustness validation of E2E-BPF three-channel multicolor imaging 11](#_Toc138982475)

[Section 6 | E2E-BPF imaging of 3D tumor spheroids 13](#_Toc138982476)

[Section 7 | Numerical robustness evaluation of E2E-BPF in multicolor imaging 15](#_Toc138982477)

[Section 8 | Aberration-informed vs. aberration-ignorant E2E-BPF designs 17](#_Toc138982478)

[Section 9 | E2E-BPF design for greater DoF extension 19](#_Toc138982479)

[Section 10 | Evaluation of photodamage and photobleaching in E2E-BPF imaging 21](#_Toc138982480)

[Section 11 | E2E-BPF design and optical microscope setup 24](#_Toc138982481)

1. Desired DoF vs. number of design variables

Consider an optical microscope with unity magnification, for simplicity. For a circular pupil function $P(\rho)$, where $\rho$ is a normalized radial coordinate, point- spread function (PSF) for a fluorescent particle can be expressed as^1-3^:

| ${h\left( \nu,\psi\right)=\left\vert U\left( \nu,\psi\right) \right\vert}^{2}$with $U\left( \nu,\psi\right)=2\int_{0}^{1} P\left( \rho\right)J_{0}\left( \nu\rho\right)\exp\left( -i2\pi\psi\rho^{2} \right)\rho d\rho$ | (S1) |
| --- | --- |

Here, $\nu$ is radial optical coordinate defined as $\nu=(2\pi/\lambda)rNA$ with the wavelength $\lambda$, the radial coordinate in the image plane $r^{2}={x^{'}}^{2}+{y^{'}}^{2}$, and the numerical aperture $NA$. $J_{0}$ is Bessel function of the first kind of order zero. $\psi$ is the defocus parameter for the particle at $z$ defined as $\psi=(z/\lambda)\cdot(NA^{2}/2n_{medium})$. One can see from **Eq. (S1)** that $U(\nu,\psi)$ is the Hankel transform of the pupil function modulated by defocus function. Introducing $t=$ $\rho^{2}$, the on-axis amplitude at $\nu=0$ can be re-written as:

| $U\left( \nu=0,\psi\right)=\int_{0}^{1} P\left( t \right)\cdot\exp(-i2\pi\psi t)dt$ | (S2) |
| --- | --- |

**Eq. (S2)** indicates that $U\left( \nu=0,\psi\right)$ is the Fourier transform of the function $P(t)$, i.e.,
$U\left( \nu=0,\psi\right)\mathcal{=F[}P(t)]$, with the Fourier transform operator $\mathcal{F}$. Using this relationship, one can easily obtain

| ${h\left( \nu=0,\psi\right)=\left\vert U\left( \nu=0,\psi\right) \right\vert}^{2}=\mathcal{F}^{*}\left[ P\left( t \right) \right]\mathcal{\cdot F}\left[ P\left( t \right) \right]\mathcal{=F}\left[ P^{*}\left( -t \right)\bigotimes P\left( t \right) \right]$ | (S3) |
| --- | --- |

where $\bigotimes$ denotes the convolution operator, and the superscript * represents complex conjugate. Hence, according to the Fourier relationship and sampling theorem, the frequency bandwidth of $h\left( \nu=0,\psi\right)$is determined by the passband supported by $P^{*}(-t)\bigotimes P(t)$, which is twice that of $P(t)$, and the sampling rate in the pupil domain determines the axial range of $h\left( \nu=0,\psi\right)$.

Now, suppose that one wishes to examine and optimize the response of $h\left( \nu=0,\psi\right)$over the range from $-\psi_{desired}$ to $\psi_{desired}$. For this, based on the sampling theorem, the sampling rate in the pupil domainshould be larger than $8\psi_{desired}$. This condition can be expressed as:

| $\frac{1}{\delta t}=\frac{2}{N_{t}}<\frac{1}{4\psi_{desired}} \text{or} N_{t}>{8\psi}_{desired}$ | (S4) |
| --- | --- |

Here, $\delta t$ and $N_{t}$ are the sampling period and the number of sampling points in the $t$ domain, respectively. Note that numeric 2 in the numerator in **Eq. (S4)** results from size of the passband in the pupil plane defined by $P^{*}\left( -t \right)\bigotimes P\left( t \right)$. On the other hand, it should be noted that uniform (or equidistant) sampling in the $t$=$\rho^{2}$ domain leads to nonuniform sampling in the $\rho$ domain, with sampling frequencies increasing with larger $\rho$. The smallest sampling period (or the highest sampling rate) in the $\rho$ domain is found to be $\delta\rho$= $\delta t/2$ at $\rho$= 1. We therefore sampled the $\rho$ space uniformly with this sampling $\delta\rho$, which also results in $N_{t}$ points in the $\rho$ space (i.e., $K=N_{t}$).

In our study, we set the desired DoF to be 16× that of clear aperture, which corresponds to $\psi_{desired}$ = 5.4. Using the criterion in **Eq. (S4)**, the number of sampling points $K$ should be larger than 43.2 in the $\rho$ domain. To validate our analysis, we performed the E2E-BPF design with different ring numbers $K$ (**Fig. S1**) for the desired DoF of 16× that of clear aperture (i.e., $\psi_{desired}$= 5.4). The results revealed that designs with more than 64 rings produced the same designs. On the other hand, the designs with less than 64 rings could not yield E2E-BPF with the desired performance.


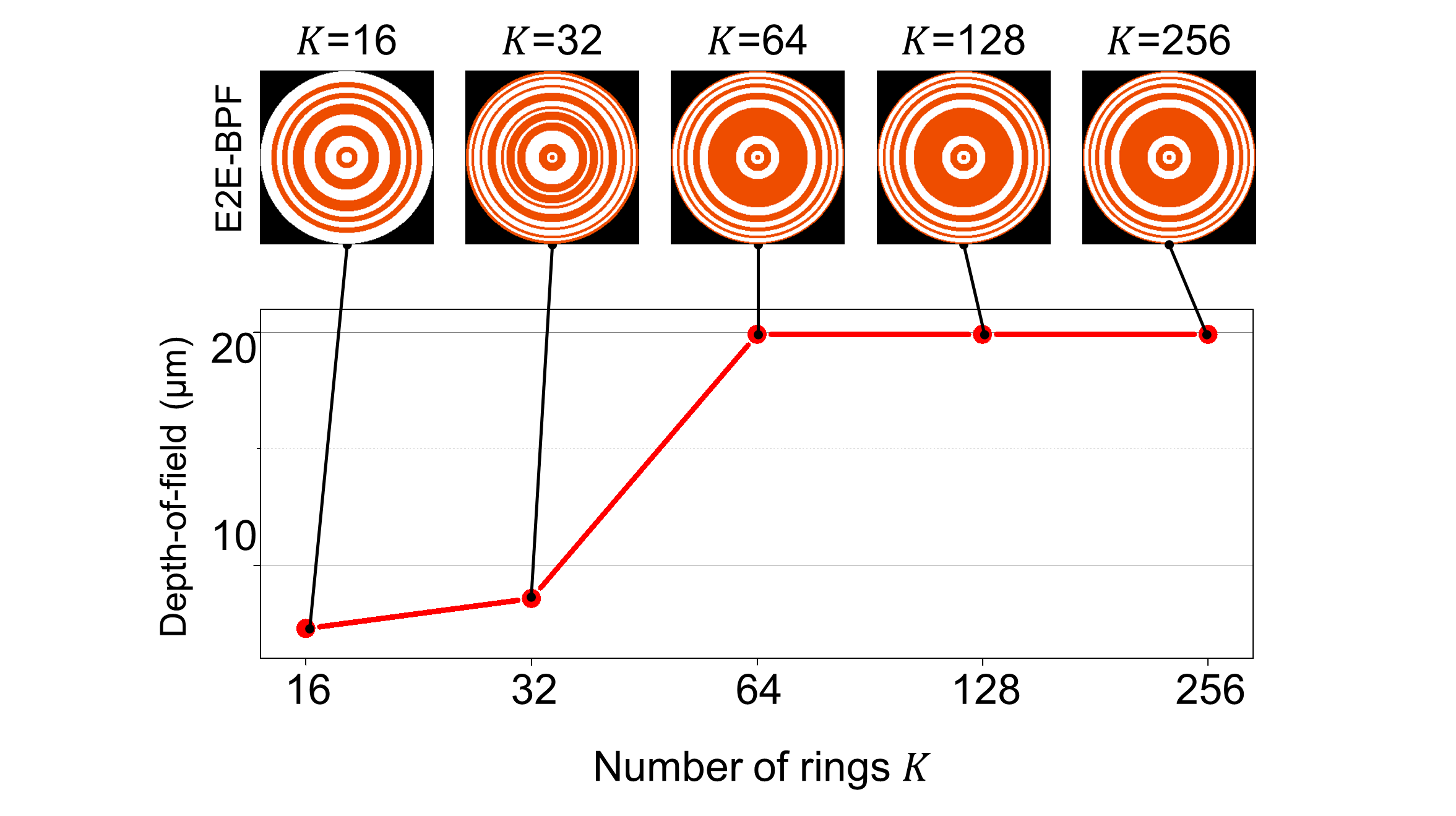


Fig. S1. DoF extension performance of E2E-BPFs designed with various numbers of rings ($\boldsymbol{K}$). It is observed that the designs more than 64 rings produced the same designs with desired performance.

1. Evaluated imaging performance of various E2E-BPF structures designed with different initial conditions

Numerical simulations were performed to investigate DoF-extension performance of the E2E-BPF structures designed under various initial conditions. We considered three axi-symmetric phase functions, i.e., phase axicon, random phases with a normal distribution of zero mean and a standard deviation of 0.8, and spherical aberration, as the initial conditions, and jointly optimized E2E-BPF structures and reconstruction networks. We then used 820 images in the dataset^4^ to evaluate the imaging performance of each design based on the RMSE and SSIM values as a function of defocus distances. The results are summarized in **Supplementary Table 1 and Fig. S2**. We determined the DoF enhancement factor by dividing the DoF for pupil A ($\mathrm{DoF}_{A}$) by the DoF of the clear aperture ($\mathrm{DoF}_{\mathrm{clear}}$). It can be observed that the E2E-BPF designed with phase axicon offers superior imaging performance to other BPF designs.

| **Supplementary Table 1 \| Compared imaging performances of the E2E-BPFs designed with various initial conditions.** | | | | |
| --- | --- | --- | --- | --- |
| **Initial condition** |  | **RMSE** | **SSIM** | $\mathbf{DoF}_{\mathbf{A}}$**/**$\mathbf{DoF}_{\mathbf{clear}}$ |
| Phase axicon  ($exp(i10\rho)$) |  | **0.015±0.006** | **0.946±0.020** | **16.74** |
| Random noise  ($mean=0$,$s.d.=0.8$) |  | 0.028±0.018 | 0.906±0.078 | 4.47 |
| Spherical aberration  ($exp(i10\rho^{3})$) |  | 0.030±0.014 | 0.901±0.071 | 10.21 |
| Clear aperture (without U-Net) |  | 0.056±0.034 | 0.766±0.127 | 1.00 |


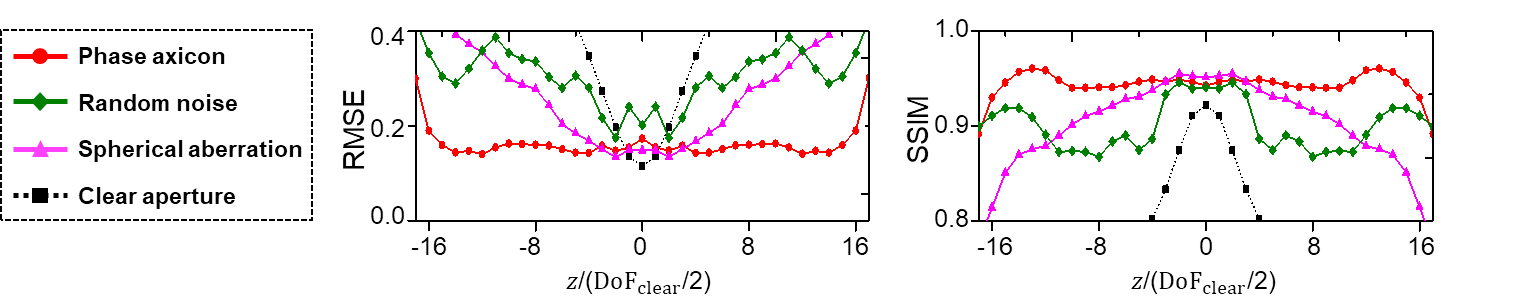


Fig. S2. Compared imaging performances (i.e., RMSE and SSIM values) of the E2E-BPF filters designed with phase axicon, random phases in the range of -π to π, and spherical aberration as the initial conditions. For reference, the responses from the standard microscope (clear aperture) were also presented. The RMSE and SSIM values were evaluated over randomly permuted 820 images from the test dataset^4^ (used in Fig. 3 of the main text, N = 820).

1. Numerical evaluations on the imaging performance of E2E-BPF against reference pupil designs

We examined the imaging performance of our E2E-BPF microscope against other phase filters, which included a conventional microscope (with clear aperture) and other prior pupil filters designed for DoF-extension (**Table 1** and **Fig. S3)**. For evaluation, we used the same dataset^4^ used in **Fig. 3** of the main text to train the reconstruction U-Nets for each configuration and computed the RMSE and SSIM values at various defocus positions. The results are summarized in **Table 1**.

**Supplementary Figure 3** presents representative images obtained with various phase filters. The values below the images denote the corresponding RMSE/SSIM values. It can be noted that the E2E-BPF microscope produced high-contrast, high-resolution images at up to 16 normalized defocus distances ($z/\mathrm{Do}F_{\mathrm{clear}}$), while other pupil designs exhibited a marked decrease in terms of image quality as the defocus distance increased.


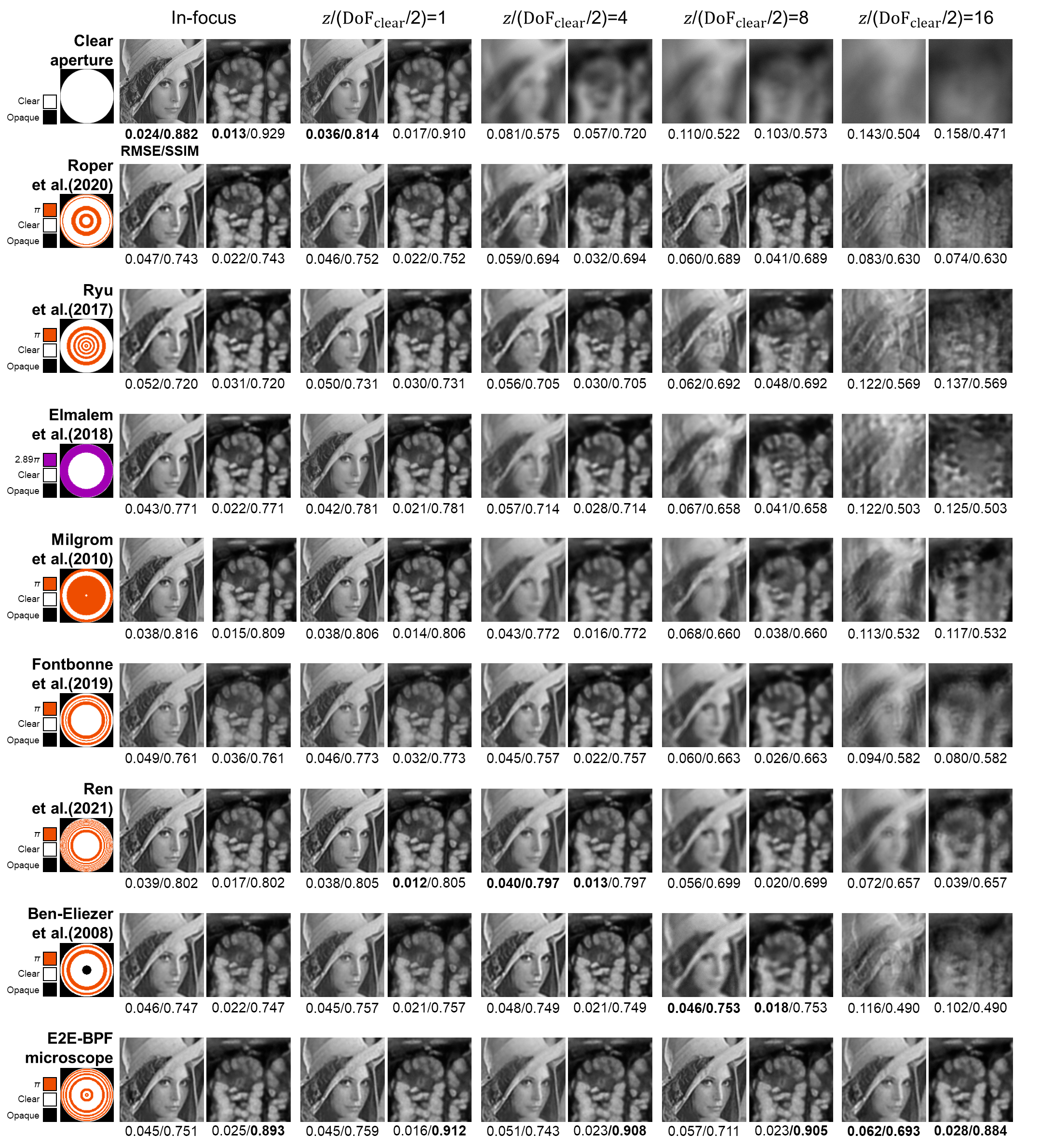


Fig. S3. Imaging performance comparisons of E2E-BPF against reference pupil designs. The images of Lenna and mouse intestine tissue section were used as the reference, and numerically imaged by a microscope equipped with various pupil filters. Note that the images were post-processed via the corresponding U-Nets optimized for each pupil design. We evaluated RMSE and SSIM values at the representative normalized axial defocus distance. The numerical values below each image denote RMSE/SSIM values.

1. Learning-based implementation of E2E-BPF filter and reconstruction U-Net

## Learning-based design of E2E-BPF microscope

The E2E-BPF design and reconstruction algorithms were implemented in Python using TensorFlow library. The first step in the algorithm is to discretize the pupil domain and set a desired DoF ($\psi_{max}$), followed by the initialization of the penalty parameter $\alpha$and the phase filter parameter $\bar{\phi}$. For our prototype, the DoF of the conventional microscope was calculated as ~1.19 μm. We nominally set our desired DoF to be 20 μm and divided the depth range into 41 segments. Within each depth segment, we assumed that PSF is identical over the 3D space. An axi-symmetric function, such as a phase axicon, was used to initialize the phase filter, which was then interpolated using the penalization function and applied to the forward imaging model to generate PSF and intermediate images. Then, the loss function was computed, and the values $\bar{\phi}$and $\mathcal{W}_{\boldsymbol{net}}$ were then updated using the built-in optimizer (Adam optimizer^5^). This is followed by an update of the penalty parameter $\alpha$. The algorithm was set to terminate if the phase values converge to the binary values (i.e., 0 or π) or maximum number of epochs was reached (10). Details of the optimization procedures are summarized in **Supplementary Table 2.**

## Image reconstruction U-Nets

We employed the U-Net architecture for image deconvolution for its end-to-end property, which allows for efficient reconstruction with no additional calculations and no need of certain forward model. The network architecture and channel information are summarized and illustrated in **Supplementary Table 3** and **Fig. 2.**

To begin, the input image is padded (44, 44, 0) by the reflection method. In the encoder, batch-normalization and the ReLU function follow each of the two 2D convolution layers after max-pooling. In the decoder, a similar architecture of batch normalization and the ReLU function were performed in each convolutional layer. The skip connection was concatenated to each convolutional layer after every up-sampling step. The batch normalization and dropout with 30% rate were also deployed during U-Net training. With five max-pooling steps, the convolutional layers of the encoder start from (3×3, 1) to (3×3, 32), at last to (3×3, 512). The decoder steps are similar to those of the encoder, except that the layers are reversed and concatenated to each layer. In the last stage, applying a 1×1 convolution with the tanh activation function yields an output by adding the input image. The padding operation in the input stage is removed at the end of the network.

## Supplementary Table 2 | Joint optimization of E2E-BPF and image reconstruction U-Nets.

| Algorithm $E2E-BPF$ | | |
| --- | --- | --- |
| 1: | $\mathbf{Procedure}E2E-BPF(\mathcal{N},K,\psi_{desired} )$ | Reconstruction network, number of rings, desired DoF |
| 2: | $epoch=0;\alpha=0$; $\bar{\phi}_{0}=AXICON(\psi_{desired})$ | Initialize BPF with a phase axicon |
| 3: | $\mathbf{repeat}$ |  |
| 4: | $I=Optics\mathcal{(P}\left( \bar{\phi}_{0} \right),\psi_{desired}, I_{T})$ | Obtain intermediate image via forward imaging model |
| 5: | $\hat{I}\mathcal{=N(}I)$ | Input the intermediate to the reconstruction U-Nets to obtain output image |
| 6: | $\mathcal{L}=RMSE\left( I_{T},\hat{I} \right)+\alpha(\nabla\mathcal{P)}$ | Evaluate loss function |
| 7: | $Compute \nabla\mathcal{L}$ | Backward propagation |
| 8: | $\mathcal{W}_{\mathbf{net}}= \mathcal{W}_{\mathbf{net}}+\Delta\mathcal{W}_{\mathbf{net}}(\partial L/\partial\mathcal{W}_{\mathbf{net}})$ | Adam optimizer step |
|  | $\bar{\phi}= \bar{\phi}+\Delta\bar{\phi}(\partial L/\partial\bar{\phi})$ |  |
| 9: | $\alpha\leftarrow min(\alpha_{\max},\alpha+\Delta\alpha)$ | Increment $\alpha$ |
| 10: | $epoch\leftarrow epoch+1$ | Continuation |
| 11: | $\mathbf{until}\nabla\mathcal{P<}\epsilon_{g}^{*}$ | Check for convergence |
| 12: | $\mathbf{end} \mathbf{procedure}$ |  |

- We set E2E-BPF optical parameters to be $NA$ = 0.75, pixel size = 6.5 μm, $M$ = 33.3, $\lambda$ = 525 nm, $n_{medium}$ = 1, and desired DoF as $\psi_{desired}$ = 5.4.
- The number of rings $K$ was set to 64.
- The Adam optimizer was trained with a batch size of 41, learning rate of 1e-8 for the phase mask and 1e-4 for the U-Net, and default exponential decay rate for both moment estimates (0.9 and 0.999, respectively)
- The $\alpha$ parameter was updated as follows: $\alpha_{0}$ = 0, $\alpha_{max}$= 100 and $\Delta\alpha$ = 0.05.
- The termination criteria were set as: $\epsilon_{g}^{*}$ = 0.001; a maximum of 10 epochs was also imposed.

## Supplementary Table 3 |Details of image reconstruction U-Nets

| Name | Type | Output size [(batch), x, y, channel] |
| --- | --- | --- |
| Input |  | 576 x 576 x 1 |
| 2x stack encoder | Conv 3x3  Batch Normalization  ReLU | 576 x 576 x 32 |
| Maxpooling | Maxpool 2x2 | 288 x 288 x 32 |
| 2x stack encoder | Conv 3x3  Batch Normalization  ReLU | 288 x 288 x 64 |
| Maxpooling | Maxpool 2x2 | 144 x 144 x 64 |
| 2x stack encoder | Conv 3x3  Batch Normalization  ReLU | 144 x 144 x 128 |
| Maxpooling | Maxpool 2x2 | 72 x 72 x 128 |
| 2x stack encoder | Conv 3x3  Batch Normalization  ReLU | 72 x 72 x 256 |
| Maxpooling | Maxpool 2x2 | 36 x 36 x 256 |
| 2x stack encoder | Conv 3x3  Batch Normalization  ReLU | 36 x 36 x 512 |
| Dropout | Dropout | 36 x 36 x 512 |
| Upsampling | Upconvolution 2x2  Concatenate | 72 x 72 x 768 |
| 2x stack decoder | Conv 3x3  Batch Normalization  ReLU | 72 x 72 x 256 |
| Upsampling | Upconvolution 2x2  Concatenate | 144 x 144 x 384 |
| 2x stack decoder | Conv 3x3  Batch Normalization  ReLU | 144 x 144 x 128 |
| Upsampling | Upconvolution 2x2  Concatenate | 288 x 288 x 192 |
| 2x stack decoder | Conv 3x3  Batch Normalization  ReLU | 288 x 288 x 64 |
| Upsampling | Upconvolution 2x2  Concatenate | 576 x 576 x 96 |
| 2x stack decoder | Conv 3x3  Batch Normalization  ReLU | 576 x 576 x 32 |
| Output | Conv 1x1  tanh | 576 x 576 x 1 |

1. Experimental robustness validation of E2E-BPF three-channel multicolor imaging

We examined the contrasts of mouse kidney tissue section images as a function of the defocus distances (**Fig. 6**). A series of grayscale images in **Fig. S4a-d** are the images of each fluorescence channel at various depth positions obtained from the standard (clear aperture) and E2E-BPF microscopes, respectively. The numerical values below the images denote the local image contrast, evaluated as $(I_{max}-I_{min})/(I_{max}+I_{min})$, where $I_{max}$ and $I_{min}$ represent the maximum and minimum pixel values, respectively. It is clearly seen that the images from the E2E-BPF microscope featured contrast values larger than 0.89 in the depth range from 0 μm to 3 μm, and the mean contrast value was measured to be 0.94. In E2E-BPF microscopy images, the structures of the glomeruli and nuclei were clearly visualized (**Fig. S4a**), and the tubules, ducts, and nuclei were resolved in various regions (**Fig. S4b-d**). In contrast, the standard microscope provided relatively low-contrast images, even though the object was placed at the focal plane of the microscope. The mean contrast was measured to be 0.82 over the range of 0 μm to 3 μm, and the contrast estimated from the focal plane images of the standard microscope was 0.88, which is smaller than the minimum contrast obtained in the images from E2E-BPF microscope.


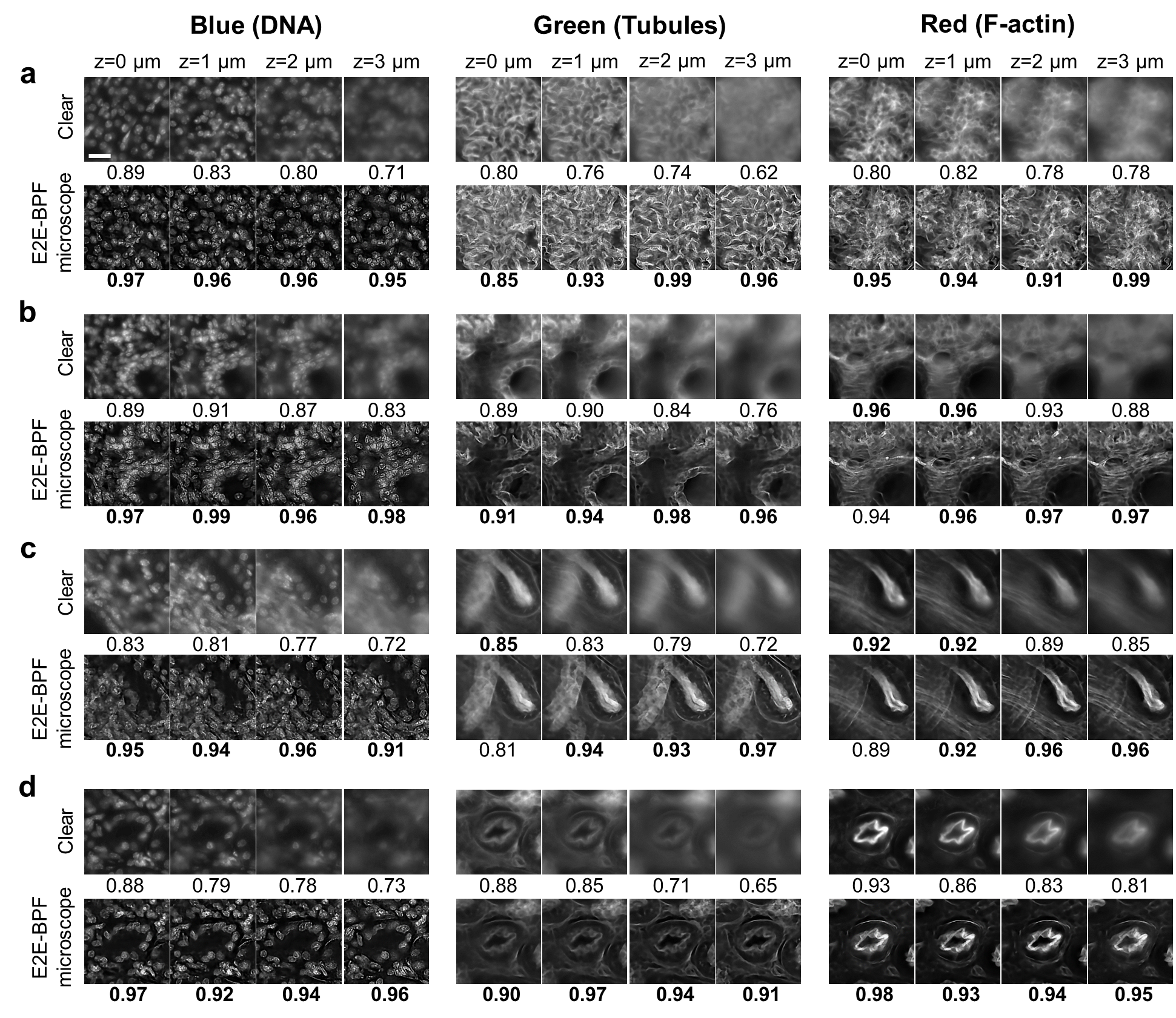


Fig. S4. Evaluated image contrasts at each fluorescence channel as a function of defocus distances. The images of (Fig. 6) in the main text were magnified and their contrast values were computed at various depth positions. Scalebar denotes 20 μm. The numeric values below the images represent the local image contrast. a, b Glomerular region, same as (Fig. 6b1, c1) and (Fig. 6 d1, e1), respectively. c, d Tubule and duct region, same as (Fig. 6b2, c2) and (Fig. 6d2, e2), respectively.

1. E2E-BPF imaging of 3D tumor spheroids

We further compared imaging performance of E2E-BPF against standard microscope by imaging 3D tumor spheroids. Two tumor spheroids were imaged with E2E-BPF and standard microscopes (**Fig. S5**). The effective $NA$ and magnification of the microscopes were identical to be 33×/0.75$NA$. The estimated sizes of the spheroids in the lateral and axial dimensions were ~300 μm and ~50 μm. Calcein AM (green) signal could be measured on the surface and inside the spheroid. The standard microscope provided images with low contrast across the entire FoV. The E2E-BPF microscope also suffered from blurs from out-of-DoF but produced the images with higher contrast (**Fig. S5**). Specifically, in the insets in **Fig. S5a1-c2**, the local contrast of the images from standard microscope were found to be 0.70 and 0.71. The local contrast of the images from E2E-BPF microscope were significantly higher, reaching 0.96 and 0.97. Similarly, in the insets in **Fig. S5b1-d2** the local contrast values of the standard microscopy images were found to be 0.67 and 0.66, whereas those in the E2E-BPF microscopy images were higher with 0.92 and 0.91, respectively.

## Sample preparation

MDA-MB-231 cells (Korea Cell Line Bank, Republic of Korea) were cultured in an RPMI 1640 (Gibco, USA) medium supplemented with fetal bovine serum (Gibco, USA) (10%[v/v]) and penicillin-streptomycin solution (Gibco, USA) (1%[v/v]). For spheroid formation, the MDA-MB-231 breast cancer cells were seeded in a U-shaped bottom well plate (CELLSTAR®, Greiner Bio-One, Austria). The cells were incubated at 37 °C for 4 days with 0.24%[v/v] methylcellulose culture medium. After forming the spheroids, the spheroids were transferred to a confocal dish (SPL, cat. no. 200350) with D-PBS medium (Gibco, USA). Immediately before imaging, cytotoxicity reagent (LIVE/DEAD ® Viability/Cytotoxicity Kit, Molecular Probes, USA) was added and incubated at 23 °C for 30 minutes to stain the spheroids.


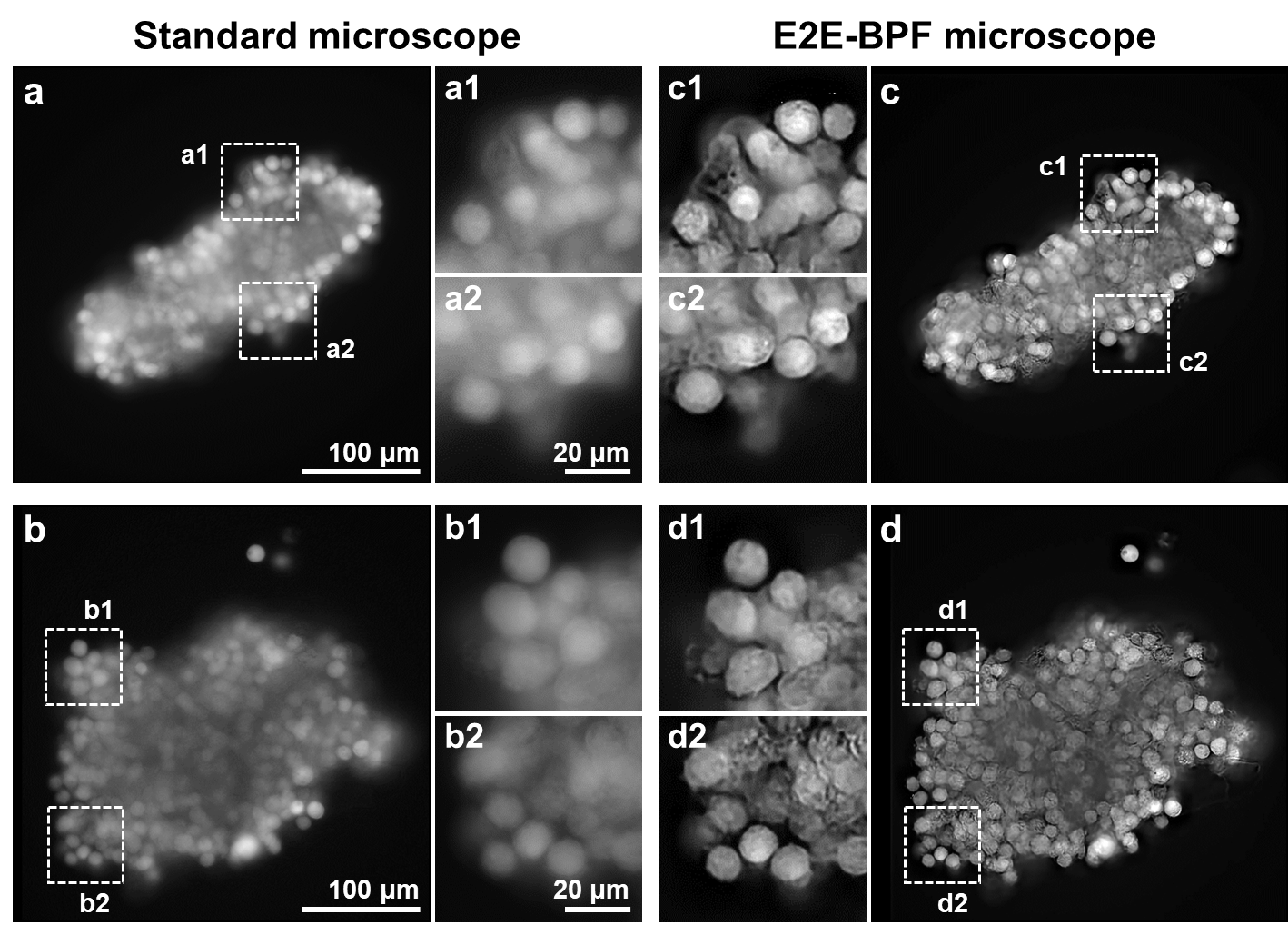


Fig. S5. Comparison of the imaging performance of a, b the standard and c, d the E2E-BPF microscopes for tumor spheroids. Selected regions of interest from each image are magnified in (a1-b2) for standard and (c1-d2) for E2E-BPF microscopes.

1. Numerical robustness evaluation of E2E-BPF in multicolor imaging

To evaluate the wavelength-dependent imaging performance of E2E-BPF, we numerically performed imaging of 820 objects labelled with various fluorescent dyes (i.e., DAPI (blue), FITC (green), TRITC (red), and Cy7 (far-red)), which exhibit different emission wavelengths. The E2E-BPF optimized at 525 nm was used to image objects from test dataset^4^ labelled with various fluorescent dyes. The results are summarized in **Fig. S6**. The E2E-BPF produce the images with high SSIM ($\geq$0.9) and small RMSE values for DAPI, FITC, and TRITC dyes. In contrast, the images of Cy7 exhibited relatively small SSIM and large RSME values.

Specifically, as shown in **Fig. S6b**, SSIM values above $\mathrm{SSI}M_{\mathrm{thr}}$(0.900) were achieved within the depths of $z$= ± 8.71 μm, ± 9.92 μm, and ± 11.2 μm for DAPI, FITC, and TRITC dyes, respectively. However, for Cy7, the SSIM value remained below the threshold over the depths ranging from -9.52 μm to 10.6 μm. The average SSIM values for DAPI, FITC, and TRITC dyes, calculated over the depths within ± 8.96 μm, were found to be 0.941, 0.946, and 0.926, respectively. In contrast, the mean SSIM for Cy7 was notably lower at 0.858. Similarly, the mean RMSE values for DAPI, FITC, and TRITC dyes, calculated over the same depths, were found to be 0.017, 0.015, and 0.022, respectively, while the mean RMSE for Cy7 was higher at 0.041.

These results indicate that E2E-BPF designed at a certain wavelength is robust to variations in emission wavelengths of <110 nm, but if the spectral shift from the design wavelength exceeds 250 nm, the performance of the E2E-BPF microscope decreases.


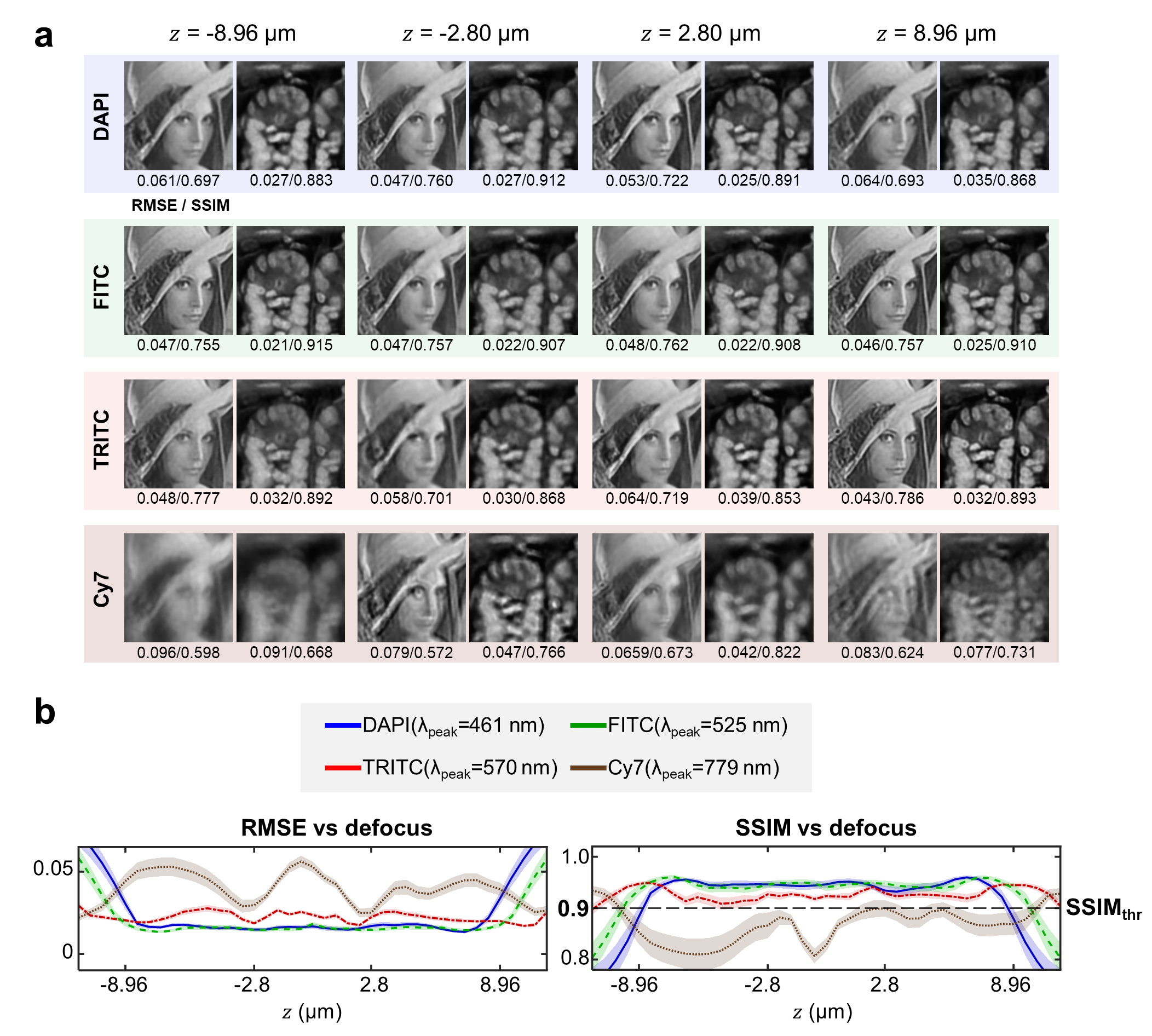


Fig. S6. Imaging performance of E2E-BPF with various fluorophores. The pictures of Lenna and mouse intestine tissue section were used as the reference, and numerically imaged by E2E-BPF and deconvolution network optimized at 525 nm. a Representative imaging results for the objects at various depth positions. The numerical values below each image denote RMSE/SSIM values. b RMSE and SSIM responses for the various fluorophores as a function of defocus distance. The solid lines represent the mean RMSE and SSIM values and the shaded areas represent standard error of the mean evaluated over randomly permuted test dataset^4^ (used in Fig. 3 of the main text, N = 820).

1. Aberration-informed vs. aberration-ignorant E2E-BPF designs

Our E2E-BPF design was performed, assuming that the microscope is aberration-free. However, in practice, aberrations are present, and this discrepancy between the design and experimental settings may contribute to the degradation of imaging performance. We conducted a numerical analysis to examine the imaging performance of E2E-BPF designed with consideration of system aberrations. The optical microscope with the same specification as our experimental platform was considered, and the system aberration was imposed as a weighted sum of the Zernike polynomials, with weights randomly assigned in the range of −5 to 5. The imposed pupil aberration and corresponding Zernike weights are shown in **Fig. S7a**. We then considered two cases; (1) BPF design informed with the system aberration, referred to as ‘aberration-informed E2E-BPF’, and (2) BPF design ignorant of system aberration, ‘aberration-ignorant E2E-BPF’. For aberration-informed design, the imposed system aberration was incorporated into physical model for imaging formation to refine E2E-BPF and deconvolution network. The designs were performed at a wavelength of 525 nm. The resultant E2E-BPF design and representative images are presented in **Fig. S7b**. It is interesting to note that both BPFs exhibit similar structures especially in the center region, but aberration-informed E2E-BPF features finer rings at the pupil edge. Both E2E-BPFs produced similar DoFs. However, the aberration-ignorant E2E-BPF suffers from severe image degradation, compared with the aberration-informed E2E-BPF. In specific, at$z$= -3.4 μm, the images from the aberration-ignorant E2E-BPF exhibited a mean SSIM of 0.901, whereas the mean SSIM value of aberration-informed E2E-BPF was measured to be 0.928. Over the depths ranging from -7.4 μm to 9.6 μm, the average SSIM values for the aberration-ignorant and aberration-informed E2E-BPFs were 0.920 and 0.934, and the mean RMSE values were found to be 0.024 and 0.017, respectively.


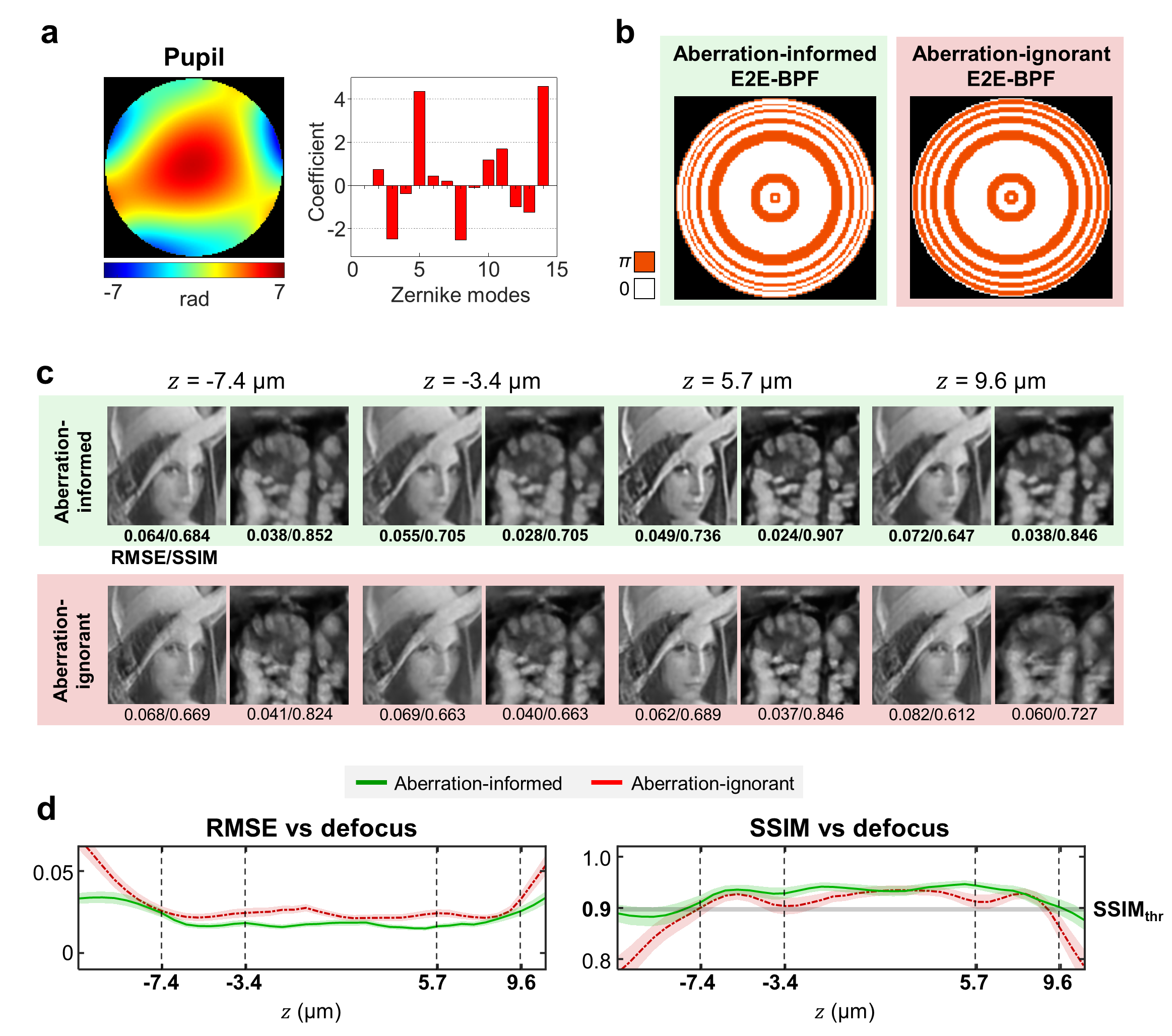


Fig. S7. Numerical imaging performance of aberration-informed and aberration-ignorant E2E-BPFs. The system aberration was imposed as a weighted sum of the Zernike polynomials, with weights randomly assigned in the range of −5 to 5. a Pupil aberration map, and corresponding weights of Zernike polynomials imposed on the E2E-BPF microscope framework. b E2E-BPF designs informed with the system aberration (aberration-informed E2E-BPF) and with no information of system aberration (aberration-ignorant E2E-BPF). c The pictures of Lenna and mouse intestine tissue section were used as the reference, and numerically imaged by a microscope equipped with the filters. The aberration map was incorporated in the image forward model. The numerical values below each image denote RMSE/SSIM values. d Imaging results for the objects at various depth positions, reconstructed with U-Nets optimized for each imaging condition. e RMSE and SSIM responses of each filter as a function of defocus distance. The solid lines represent the mean RMSE and SSIM values and the shaded areas represent standard error of the mean evaluated over randomly permuted test dataset^4^ (used in Fig. 3 of the main text, N = 820).

1. E2E-BPF design for greater DoF extension

To demonstrate the capability of our platform for further DoF extension, we performed BPF design with a desired DoF of 24× that of a clear aperture, referred to as ‘24×-DoF design’ (**Fig. S8a**). We then compared its performance against that of the original BPF design (i.e., BPF with a desired DoF of 16× that of clear aperture, ‘16×-DoF design’). For this task, we used larger number of rings (i.e., 96 rings) in the design space, which led to a design time of approximately 28 hours, which is 2.5× longer compared to the original 64-ring design (i.e., 16×-DoF design). Our design framework produced a BPF design with a significantly larger DoF of 22.08× that of clear aperture in the numerical evaluation. Although the targeted 24×-DoF extension was not fully realized, this result indicates the potential of our platform to design BPFs for further extended DoF imaging. As shown in **Fig. S8b**, SSIM values above the threshold ($\mathrm{SSIM}_{\mathrm{thr}}$=0.900) could be maintained up to $z$= ± 13.13 μm for the 24×-DoF design. For the 16×-DoF design, these values were limited to $z$= ± 9.92 μm. Over their respective extended DoF ranges, the mean SSIM values for the 24×- and 16×-DoF designs were found to be 0.927 and 0.942, respectively. In the 24×-DoF design, however, we observed a slight degradation in image resolution (e.g., in-focus plane in **Fig. S8b**). To be specific, the in-focus SSIM value for the 24×-DoF design was 0.905, while that for the 16×-DoF design was higher at 0.944.

Moreover, the reduction of fluorescence signals in the detector plane should also be considered. Since BPF generates elongated PSFs in the detector region, the energy is distributed over the depth, resulting in the decrease in the measured fluorescence signal. This feature has been studied previously^1,6^. In E2E-BPF, the jointly optimized deconvolution network denoises and processes the acquired images to generate high-resolution, high-contrast images. For low-photon images, however, the influence of various noises on the images would be significant, degrading image quality. In our study, we found that compared to the 16×-DoF design, the 24×-DoF design featured decrease of the fluorescence signal within a DoF range by 37%, which is one-twentieth of a clear aperture.


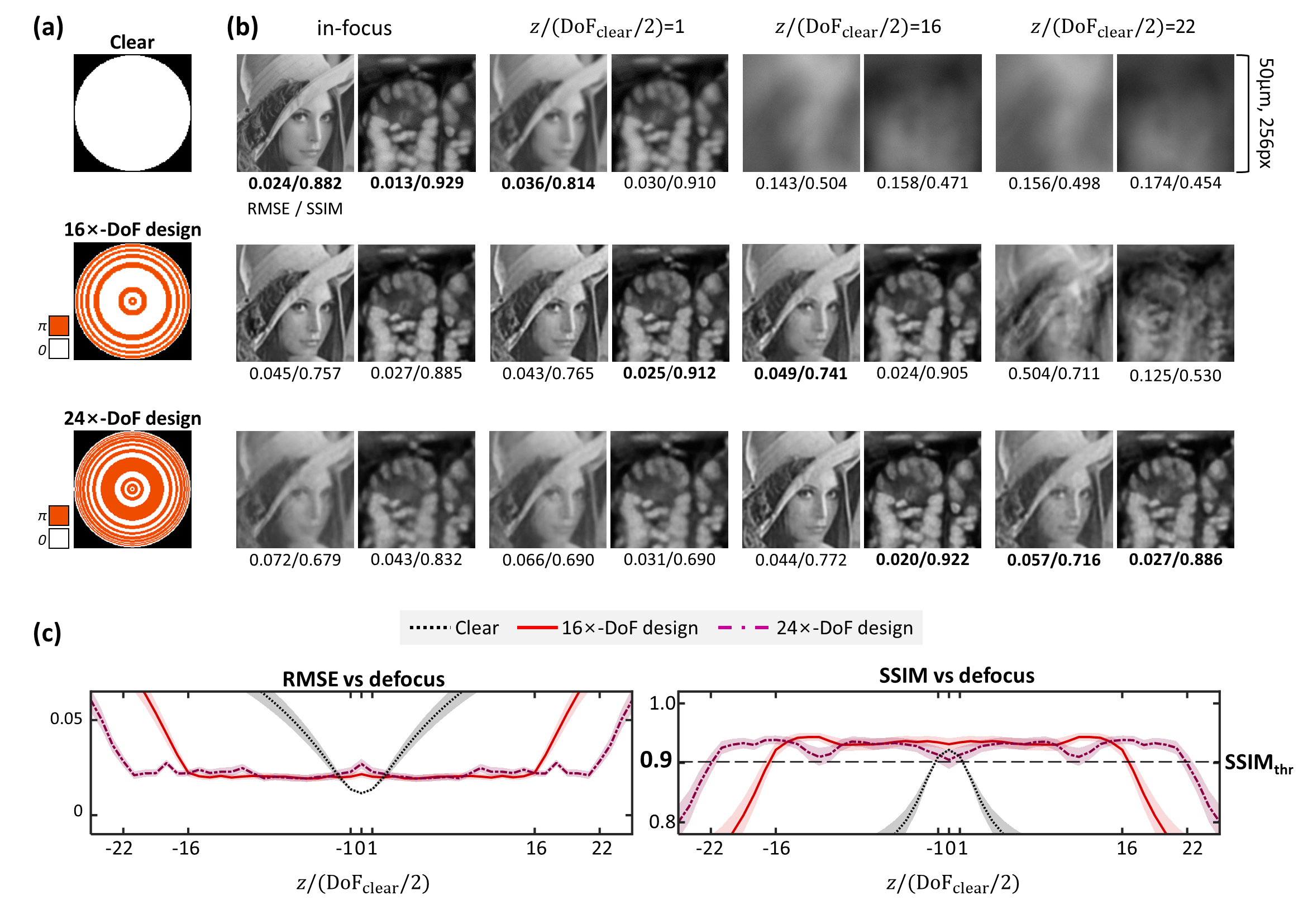


Fig. S8. Numerical imaging performance of 16×-and 24×-DoF BPF designs. The pictures of Lenna and mouse intestine tissue section were used as the reference, and numerically imaged by a microscope equipped with the filters. a BPF design results for 16x- and 24x-DoF extension. b Imaging results with 16×-DoF and 24×-DoF E2E-BPF microscopes for the objects at various depth positions. Note that the results from 16×-DoF and 24×-DoF designs were post-processed via the corresponding U-Nets optimized for each imaging condition. The numerical values below each image denote RMSE/SSIM values. c RMSE and SSIM responses of each pupil filter as a function of defocus distance. The solid lines represent the mean RMSE and SSIM values and the shaded areas represent standard error of the mean evaluated over randomly permuted test dataset (used in Fig. 3 of the main text, N=820).

1. Evaluation of photodamage and photobleaching in E2E-BPF imaging

To evaluate potential photodamage caused by E2E-BPF imaging, we performed live/dead cell counting assays on MDA-MB-231 breast cancer cells. We employed Calcein AM and Ethidium Homodimer-1 (EthD-1) as the fluorescent markers for live and dead cells, respectively. E2E-BPF imaging was conducted ten times over a 1.6 mm × 1.6 mm area at 15-minute intervals. **Fig. S9a** presents representative images captured with E2E-BPF microscope. The intensities of the illumination light were set at 0.16 mW mm^-2^ for Calcein AM and 0.24 mW mm^-2^ for EthD-1 imaging, with camera exposure times of 90 ms and 900 ms, respectively. During the 150-minute imaging experiment, the images from E2E-BPF microscope maintained consistent fluorescence signal of Calcein AM and EthD-1, as demonstrated in **Fig. S9b**. Each point represents the mean intensity of nine 2048 × 2048 images. Further evaluation of live and dead cells counting assay within FoVs of 1.6 mm x 1.6 mm revealed no discernible changes in cell viability.

We further performed longitudinal imaging of fixed mouse kidney tissue section and evaluated photobleaching in E2E-BPF imaging. The tissue sections were imaged 75 times at 2-minute intervals using E2E-BPF microscope, and the results are summarized in **Fig. S10**. One can note that the E2E-BPF does not cause any notable change in fluorescence images.

## Sample preparation

MDA-MB-231 cells (Korea Cell Line Bank, Republic of Korea) were cultured in an RPMI 1640 (Gibco, USA) medium supplemented with fetal bovine serum (Gibco, USA) (10%[v/v]) and penicillin-streptomycin solution (Gibco, USA) (1%[v/v]). To prepare a sample for evaluation of photocytotoxicity, the cultured cells were seeded at a density of 1 × 10^4^ cells per well in a µ-Slide 8 well (IBIDI, Germany) and incubated at 37 °C for 24 h. Then, the cells were washed with Dulbeco’s phosphate-buffered saline (D-PBS) (Gibco, USA). After washing the cells, each 100 μL of D-PBS and cytotoxicity reagent (LIVE/DEAD ® Viability/Cytotoxicity Kit (L3224), Molecular Probes, USA) was added and incubated for 30 minutes at 23 °C. Live cells were indicated by the conversion of Calcian AM in the cytotoxicity reagent to green fluorescent calcein as hydrolyzed by intracellular esterases. The dead cells were assessed by the red fluorescent signal generated by entering Ethidium Homodimer-1 in the reagent into the cells with compromised membranes and binding to nucleic acid.


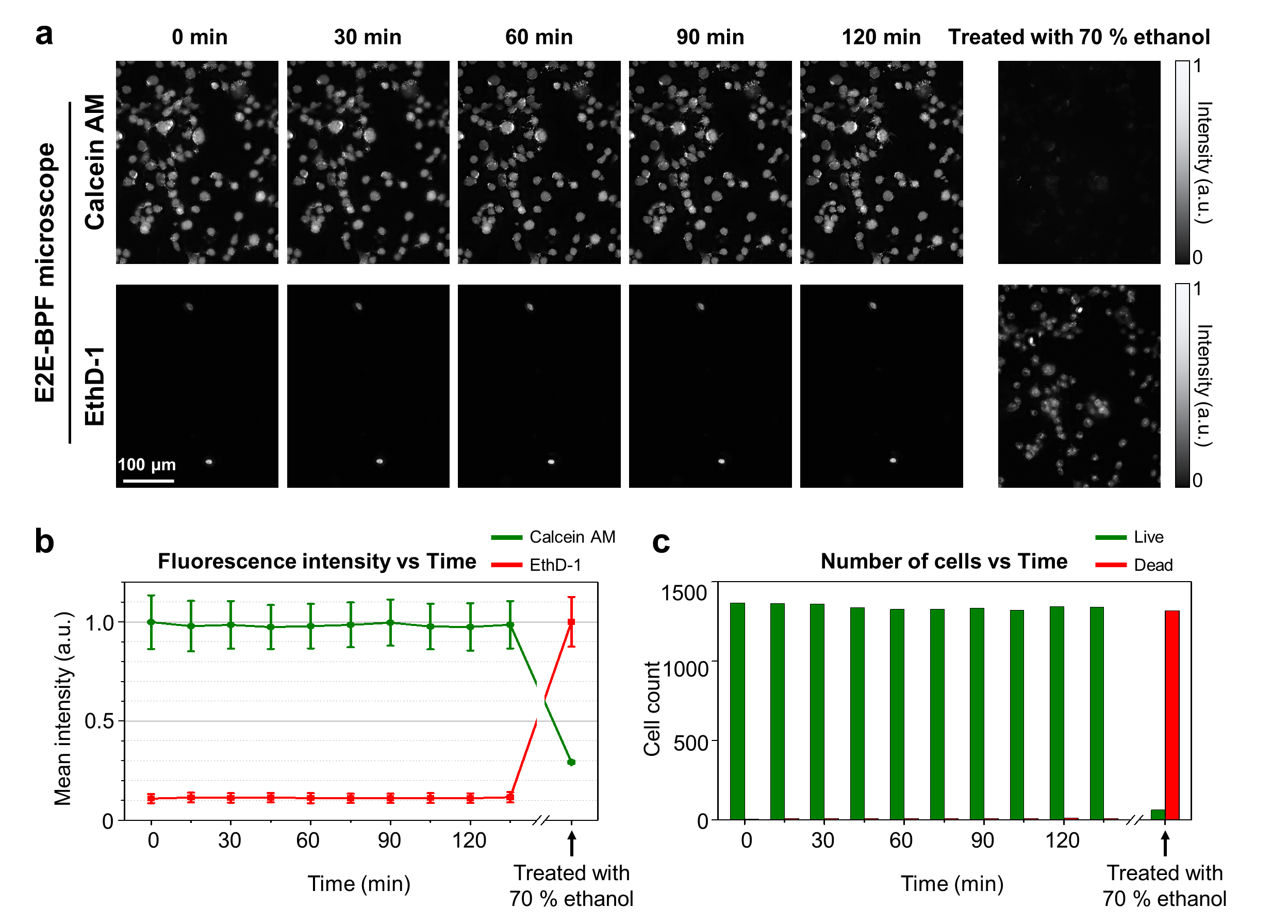


Fig. S9. Evaluated photodamage in E2E-BPF imaging. Cell viability tests were performed on MDA-MB-231 breast cancer cells. a Representative images of a region of interest imaged by E2E-BPF microscope. Calcein AM and Ethidium Homodimer-1 (EthD-1) were used as the fluorescent markers for live and dead cell imaging, respectively. Shown in the last column are the images of ethanol-treated (killed) cells, which presents significant reduction in Calcein AM signal and increase in EthD-1 signal. Scale bar: 100 μm. b Mean fluorescence signals from Calcein AM and EthD-1 channels over the 150-minute experiment. Each point represents the mean intensity of nine 2048 × 2048 images and the error bar denotes the standard deviation. The measurements were normalized to the maximum values for each case. c Number of cells in both channels over the 150-minute experiment. The E2E-BPF microscope maintained consistent fluorescence intensities throughout the experiment and did not cause any discernible changes in fluorescence images.


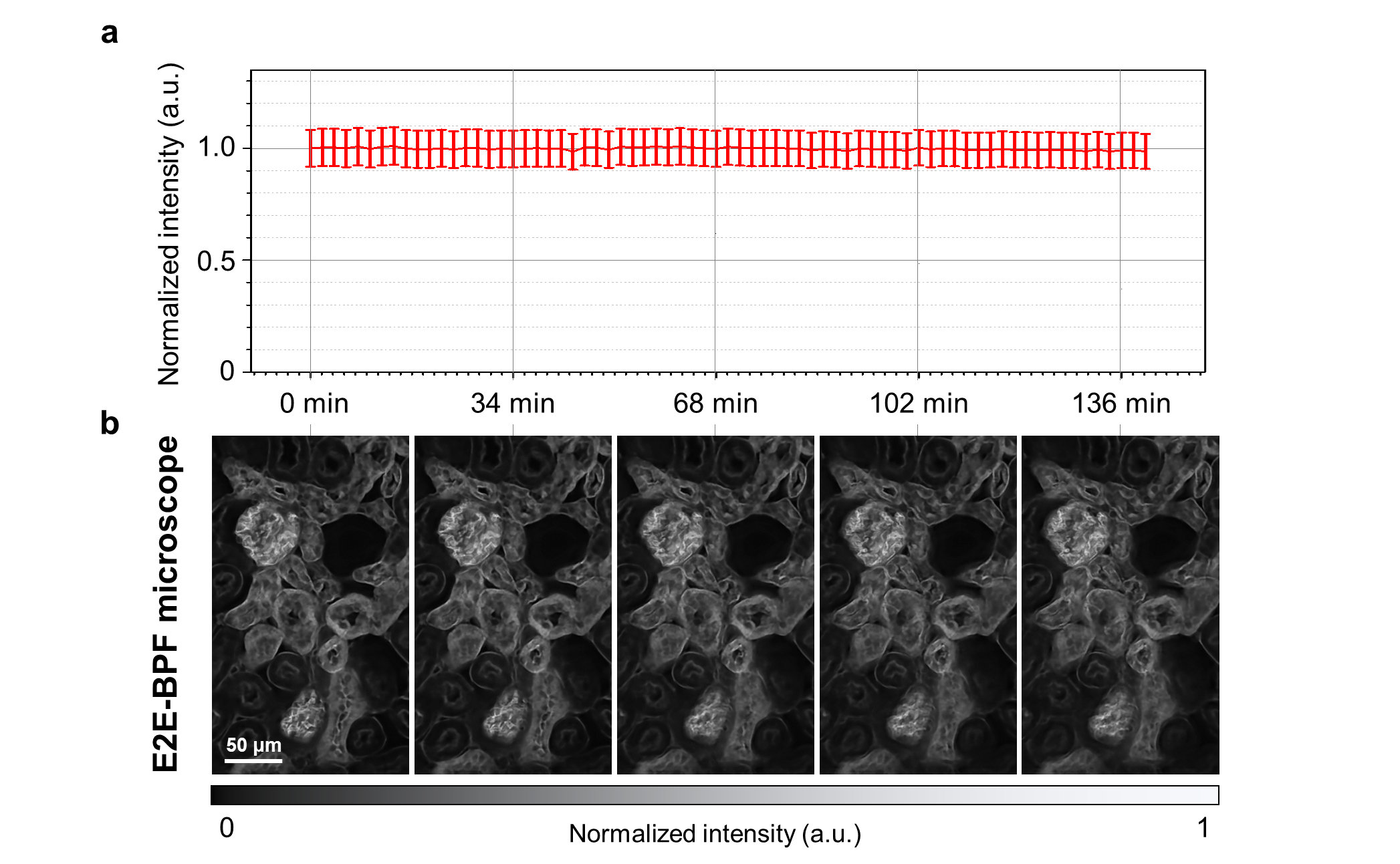


Fig. S10. Evaluated photobleaching in E2E-BPF imaging. A mouse kidney section, with a nominal thickness of 16 µm, was used for large-scale tissue imaging. Alexa Fluor 488 wheat germ agglutinin was employed to label elements of the glomeruli and convoluted tubules. The imaging was performed with excitation power of 0.16 mW mm^-2^ with camera exposure time of 90 ms. a The mean intensity evaluated over nineteen 2048 × 2048 images as a function of imaging time. The intensity was normalized to the measurement at the initial timepoint (time = 0 min). The error bars indicate the standard deviation. b Representative images of a region of interest recorded by the E2E-BPF microscope every 2 minutes over a span of 140 minutes.

1. E2E-BPF design and optical microscope setup

The E2E-BPF design and optical setup are provided in **Fig. S11**. The E2E-BPF consisted of 12 rings with alternating 0 and π phases in the aperture. In our prototype, the pupil diameter was 9 mm, and the phase transition radial coordinates $\boldsymbol{r}=(r_{1},\ldots, r_{12})$ are provided in **Fig. S11**. The E2E-BPF was fabricated through a photolithography and placed in the pupil plane of the optical system.


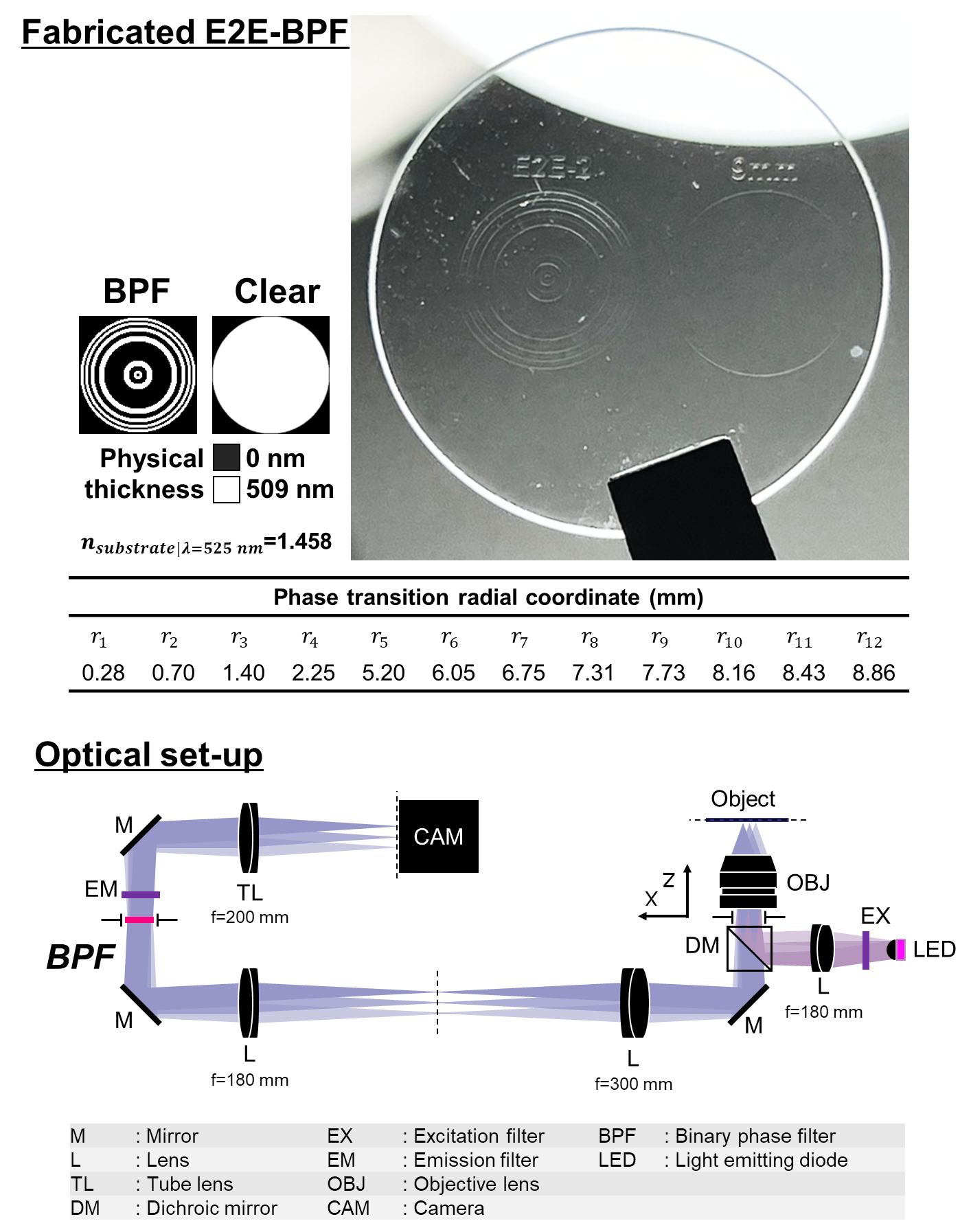


Fig. S11. Fabricated E2E-BPF filter and optical microscope set-up.

#### Reference

1 Sheppard, C. & Hegedus, Z. Axial behavior of pupil-plane filters. *JOSA A* **5**, 643-647 (1988).

2 VanderLugt, A. Optimum sampling of Fresnel transforms. *Applied Optics* **29**, 3352-3361 (1990).

3 Piestun, R., Spektor, B. & Shamir, J. Wave fields in three dimensions: analysis and synthesis. *JOSA A* **13**, 1837-1848 (1996).

4 Borkowski, A. A. *et al.* Lung and colon cancer histopathological image dataset (lc25000). *arXiv preprint arXiv:1912.12142* (2019).

5 Kingma, D. P. & Ba, J. Adam: A method for stochastic optimization. *arXiv preprint arXiv:1412.6980* (2014).

6 Ryu, S. & Joo, C. Design of binary phase filters for depth-of-focus extension via binarization of axisymmetric aberrations. *Optics express* **25**, 30312-30326 (2017).
